# Hydrophobicity Modulated Interfacial Water Distribution for Enhanced Kinetics of HER

**DOI:** 10.1002/advs.75273

**Published:** 2026-04-14

**Authors:** Yucheng Dong, Xinfa Wei, Xiangdong Xue, Hanxiao Wang, Qing Dong, Jian Liu

**Affiliations:** ^1^ College of Materials Science and Engineering Qingdao University of Science and Technology Qingdao P. R. China; ^2^ State Key Laboratory of Photoelectric Conversion and Utilization of Solar Energy Qingdao New Energy Shandong Laboratory Qingdao Institute of Bioenergy and Bioprocess Technology Chinese Academy of Sciences Qingdao P. R. China

**Keywords:** electrochemical, hydrogen evolution reaction, hydrophobicity, interfacial water

## Abstract

The interfacial water structure is crucial for the hydrogen evolution reaction (HER) performance. However, the explicit relationship between the local interfacial water structure and the HER process remains elusive. Here, we engineer a hydrophilic–hydrophobic Ni‐PTFE interface to modulate the interfacial water organization and enhance HER kinetics. Electrochemical measurements reveal that the Ni‐PTFE composite significantly reduces the HER overpotential from 36 to 19 mV at 10 mA·cm^−2^, comparable to the performance of the precious metal catalyst Pt. In situ ATR‐SEIRAS indicates that the proportion of H_2_O_(above‐gap)_ at the hydrophilic–hydrophobic Ni‐PTFE interface increased from 36.6% to 50.5%, facilitating proton transfer and water dissociation. Density functional theory calculations further demonstrated that the hydrophobic PTFE rearranges interfacial water on the Ni surface into a wave‐like distribution centered around the PTFE segments, leading to alternating water‐rich and water‐deficient regions that spatially differentiate active sites for water dissociation and H_2_ detachment, respectively. The study offers an insight into enhancing HER performance through strategic hydrophobic modification aimed at regulating interfacial water structure.

## Graphical abstract

In electrocatalysis, hydrophilic structures improve interfacial water management by forming a continuous, compact water film that facilitates proton/hydrogen transport, thereby lowering the effective adsorption free energy of H^*^. By contrast, hydrophobic structures enable rapid gas transport and promote the capture and local enrichment of gaseous intermediates/products. Integrating these phases in an ordered manner can produce a dynamic coupling: hydrophilic structures accelerate water dissociation, whereas hydrophobic structures rapidly concentrate gas‐phase species.

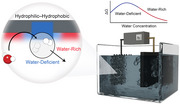

## 1 Introduction

Interfacial water molecules play a crucial role in electrocatalytic processes, with their molecular configuration and dynamics directly governing charge transfer efficiency and reaction kinetics [1, 2, 3, 4]. Recent in situ studies have revealed that water actively regulates electrocatalytic performance through its molecular orientations and hydrogen bond (H‐bond) networks, beyond its traditional roles as solvent and reactant [5, 6, 7, 8, 9]. Since interfacial water structure is highly tunable and has a profound impact on catalytic performance, these findings underscore the importance of engineering the electrode‐water interfacial structure in addition to traditional active site modifications. The hydrogen evolution reaction (HER) is a critical process for sustainable hydrogen production [10, 11, 12, 13, 14, 15, 16]. Understanding interfacial water structure is essential for HER optimization, as the electrode–electrolyte interface dictates proton availability, charge transfer, and reaction energetics. In a seminal study, Li and Pan utilized in situ surface‐enhanced Raman spectroscopy to identify two structurally distinct water populations at the Pd electrode–electrolyte interface: conventional hydrogen‐bonded (H‐bonded) water and cation‐coordinated water. Importantly, cation‐coordinated water assembles into highly ordered interfacial structures at the electrode surface through cation coordination under applied negative potentials, thereby enhancing charge transfer efficiency and HER kinetics [17]. These insights motivate various regulation strategies ranging from atomic‐scale coordination engineering to mesoscale electric double‐layer manipulation [18, 19, 20, 21]. Beyond these approaches, the balance between hydrophilicity and hydrophobicity at the electrode surface offers another powerful yet underexplored lever for controlling interfacial water architecture.

**SCHEME 1:**
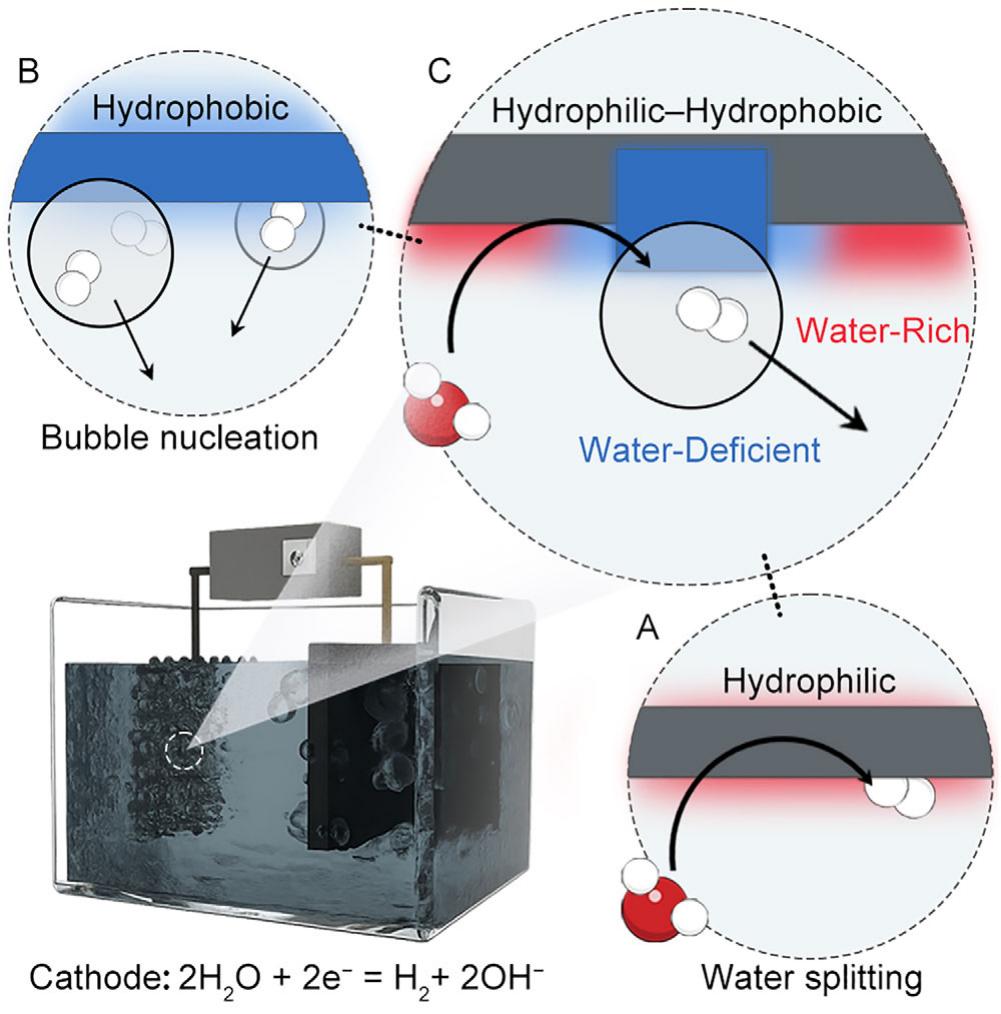
The design concept of the hydrophilic–hydrophobic structure.

Surface wettability has been widely acknowledged as an important factor in electrocatalysis [22, 23, 24, 25, 26]. However, hydrophobic materials have been conventionally dismissed for water splitting due to concerns over elevated interfacial resistance and electrolyte dewetting [27, 28, 29, 30, 31]. This perception has confined surface wettability engineering to superhydrophilic approaches, largely overlooking the potential of hydrophobic materials. This view requires reconsideration in light of mechanistic insights from other electrocatalytic reactions. At hydrophilic surfaces (Scheme 1A), interfacial water assembles into densely H‐bond networks that facilitate proton transport and accelerate water dissociation, but hinder bubble nucleation and detachment [32, 33, 34]. Conversely, at hydrophobic surfaces (Scheme 1B), disrupted interfacial water configurations promote gas molecule capture and enrichment and enable facile bubble nucleation and detachment, but suffer from reactant depletion [35, 36, 37]. Critically, the rational integration of hydrophilicity and hydrophobicity offers a strategy to overcome these constraints through the synergistic coupling of distinct water structures (Scheme 1C): hydrophilic domains provide structured water environments for efficient water activation, while hydrophobic domains create low‐density water regions that enable facile gaseous product nucleation and detachment. Accordingly, to exploit the potential of hydrophobic components in HER, rational catalyst design must engineer the spatial distribution of hydrophilic and hydrophobic regions to optimize interfacial water architecture, thereby improving both reactant activation and product removal.

Herein, we demonstrate that modulating the local hydrophobicity of hydrophilic electrode is an effective strategy for boosting the HER kinetics. We show that incorporating PTFE into hydrophilic Ni electrode can markedly lower the onset potential and Tafel slope. By combining electrochemical testing, in situ spectroscopic analysis, and theoretical modeling, we systematically investigate the impact of hydrophobicity on interfacial water structure. Density functional theory (DFT) calculations reveal that local hydrophobic domains modulate the adsorption behavior of interfacial water and its dissociation intermediates. Furthermore, by combining in situ attenuated total reflection surface‐enhanced infrared absorption spectroscopy (ATR‐SEIRAS) with molecular dynamics (MD), we elucidate how PTFE‐induced hydrophobicity reorganizes the interfacial water network and thereby enhances HER activity on Ni surfaces. Our findings highlight that hydrophobic engineering plays a pivotal role in regulating the interfacial water distribution and optimizing adsorption free energies, ultimately boosting HER performance.

## 2 Results

### 2.1 Catalyst Structural Characterization

**FIGURE 1:**
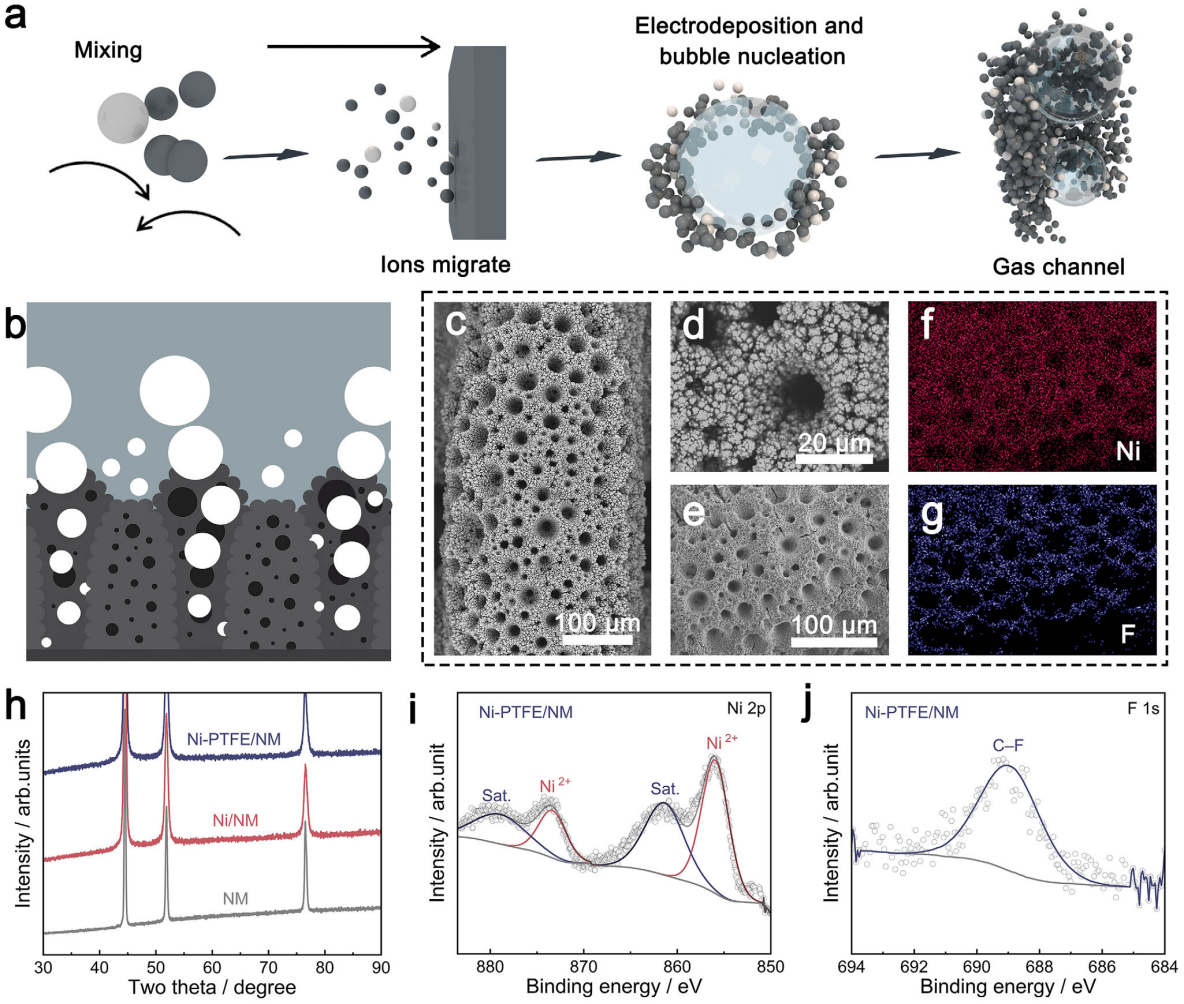
Structural characterization of Ni‐PTFE/NM electrode. (a) Schematic illustration of bubble‐templating electrodeposition for catalyst layer fabrication. (b) Scheme image and (c‐d) SEM image of Ni‐PTFE/NM electrode. (e‐g) EDS mapping of Ni and F. (h) The XRD pattern of Ni‐PTFE/NM, Ni/NM, and NM electrodes. High‐resolution XPS (i) Ni 2p and (j) F 1s spectra of Ni‐PTFE/NM electrode.

To fabricate the Ni/PTFE composite catalyst, we employed a one‐step co‐electrodeposition method to simultaneously deposit Ni and PTFE onto nickel mesh (NM) substrates (Figure 1a). During cathodic polarization, the HER occurs simultaneously with Ni deposition, producing H_2_ bubbles that continuously nucleate, grow, and detach from the electrode surface. These bubbles acted as dynamic sacrificial templates, physically obstructing the lateral spreading of the depositing Ni layer and creating interconnected macropores (Figure 1b). Scanning electron microscopy (SEM) images of the catalyst layer reveal that, compared to the smooth NM electrode surface (Figure S1a,b), the Ni/NM electrode surface exhibits interconnected gas channels and a 3D porous architecture (Figure S1c,d).

Cetyltrimethylammonium bromide (CTAB), with its cationic charges imparted by its molecular structure, creates electrostatic repulsion among particles, thereby preventing PTFE agglomeration [38]. Concurrently, upon application of an electric field, this cationic surfactant facilitates the charging of PTFE and promotes its adsorption on the electrode surface [39]. The Ni‐PTFE co‐deposited catalyst layer exhibits a microstructure with a decrease in structural density and an increase in channel size due to the introduction of PTFE (Figure 1c,d; Figure S2a,b). This phenomenon is likely attributed to the disruption of the coherent growth of the Ni catalyst layer caused by PTFE embedding. The elemental mapping confirms the uniform distribution of Ni and F within the deposited layer, with the energy‐dispersive X‐ray spectroscopy (EDS) analysis indicating an atomic ratio of 2.5% for F element (Figure 1e–g; Figure S2c,d). As shown in Figure S3, the size of the channels is tunable by varying the amount of incorporated PTFE (Table S1). Additionally, the PTFE content varies with its loading amount (Figure S2c,d; Figures S4 and S5).

Transmission electron microscopy (TEM) was employed to investigate the morphology and microstructure of the catalyst layer. The Ni‐PTFE/NM sample exhibits a layered, stacked architecture (Figure S6a,b), consistent with the sheet‐like morphology observed by SEM (Figure S2b). Compared with the Ni/NM electrode (Figure S7a,b), the Ni‐PTFE composite shows a thinner and more dispersed structure, indicating that PTFE suppresses the continuous growth of Ni during electrodeposition. High‐resolution TEM (HRTEM) images reveal lattice fringes with a spacing of 0.20 nm in both samples, corresponding to the Ni (111) crystal plane (Figure S6c,d; Figure S7c,d). Notably, abundant pores are visible on the surfaces and within the interiors of the sheets, indicating the high porosity of the catalyst. EDS elemental mapping confirms the successful introduction of PTFE with Ni and F signals uniformly distributed throughout the catalyst layer (Figure S6e–g). The PTFE content significantly influences its distribution on the Ni surface. At low PTFE loadings, fluorine appears as isolated, point‐like signals with sparse coverage (Figure S8). As the PTFE loading increases, the coverage becomes denser over the surface and edge sites (Figure S9a–e). Meanwhile, excessive PTFE loading leads to local aggregation and thick film formation (Figure S9f–h), which introduces additional charge transfer resistance and blocks active sites, thereby impairing catalytic performance.

To elucidate the chemical characteristics of the catalysts, we conducted characterizations using X‐ray diffraction (XRD), X‐ray photoelectron spectroscopy (XPS), and ATR‐SEIRAS. XRD analysis shows distinct diffraction peaks, which can be assigned to the crystallographic reflections of metallic Ni (PDF#04‐0850) (Figure 1h). No PTFE diffraction features are detected, attributable to the low PTFE loading and the overwhelming intensity of Ni reflections. XPS assessments of the Ni/NM electrode surface indicate that surface Ni exists primarily in the Ni^2+^ state at 855.8 eV (Figure S10a) [40, 41]. After PTFE co‐deposition, the Ni 2p binding energy remains essentially unchanged, suggesting that PTFE neither forms strong chemical bonds with Ni nor substantially perturbs its electronic environment (Figure 1i). The Ni‐PTFE/NM electrode exhibits a prominent F 1s peak at 688.9 eV (Figure 1j) [42] and a characteristic C 1s peak at 291.6 eV attributable to C─F bonds (Figure S10b) [43], confirming the successful co‐deposition of PTFE with Ni. In contrast, the Ni/NM electrode does not exhibit any characteristic peaks for C─F bonds (Figure S10c). ATR‐SEIRAS spectra (Figure S11) reveal two characteristic absorption bands at ∼1200 and ∼1150 cm^−1^, assignable to the symmetric (ν_s_) and antisymmetric (ν_as_) C─F_2_ stretching modes, respectively [44, 45, 46]. Notably, the integrated intensities of these bands increase systematically with PTFE loading. In summary, the primary role of PTFE pertains to enhancing hydrophobicity, porosity, and gas diffusion, rather than directly modulating the electronic structure or intrinsic catalytic activity of Ni.

### 2.2 Electrocatalytic Activity

**FIGURE 2:**
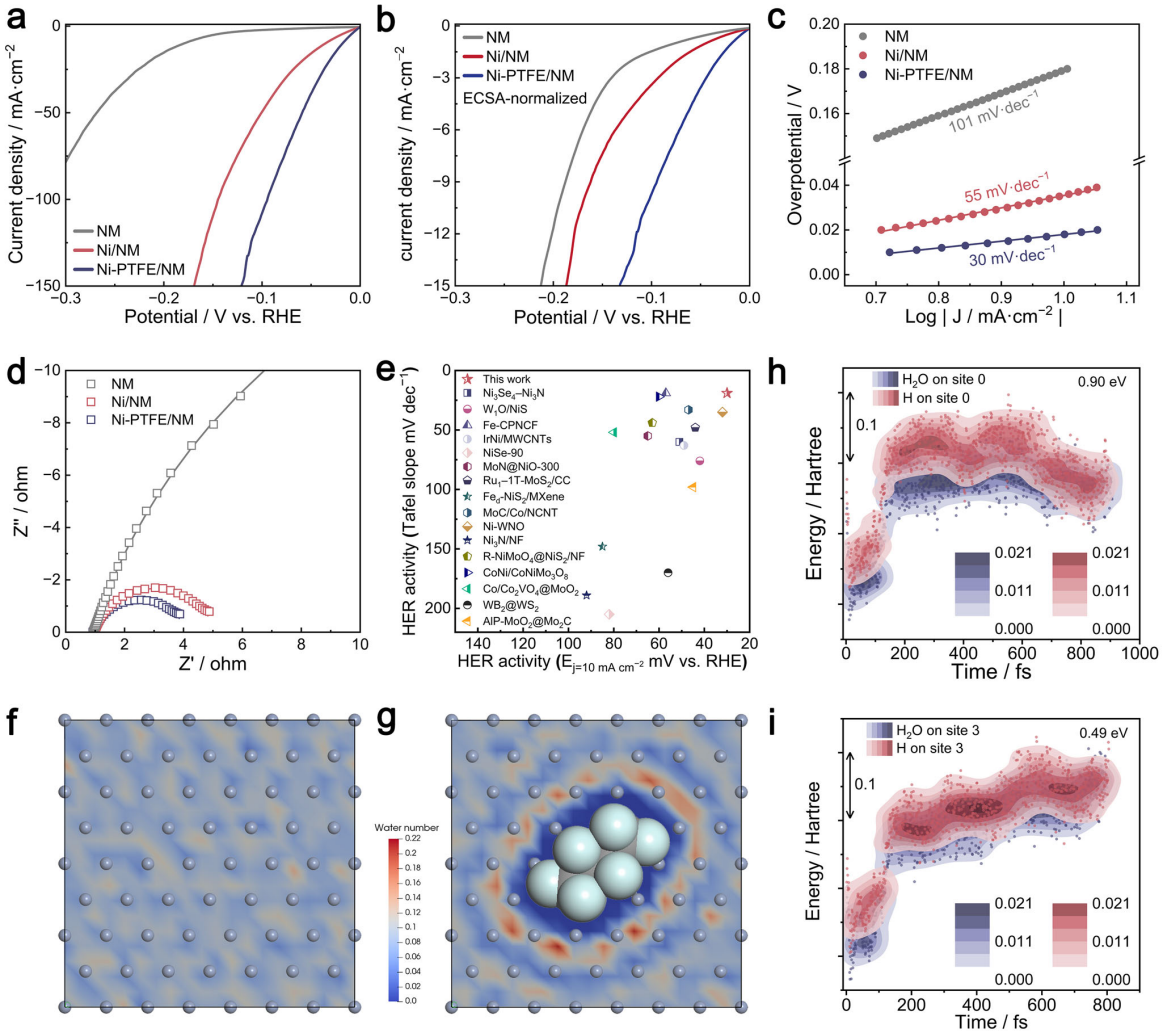
Electrochemical performance and DFT analysis of interfacial water on Ni‐PTFE. (a) HER polarization curves. (b) ECSA normalized HER polarization curves. (c) Tafel plots. (d) Nyquist plots. (e) Performance comparison of Ni‐PTFE/NM electrode with advanced non‐precious metal electrocatalysts reported in the recent literature (2025) (The detailed benchmark metrics and corresponding references are listed in Table S3). Water molecular number density distribution of (f) Ni and (g) Ni‐PTFE. The adsorption free energy distribution of H and H_2_O at (h) site 0 and (i) site 3.

Linear sweep voltammetry (LSV) curves for the electrodes were recorded in 1 M KOH at room temperature (25 °C) with a scan rate of 5 mV·s^−1^ (Figure 2a; Figure S12). At a current density of 10 mA·cm^−2^, the Ni‐PTFE/NM electrode exhibits an overpotential of 19 mV, only marginally higher than the 10 mV for Pt/C. This indicates that, in the low‐current‐density regime, the Pt/C electrode has a slightly lower onset potential than the Ni‐PTFE/NM electrode. However, at 50 mA·cm^−2^, the overpotential of the Ni‐PTFE/NM electrode (60 mV) is substantially lower than those of the NM, Ni/NM, and Pt/C electrodes. The divergence at higher current density primarily arises from electrode‑specific mass‑transport characteristics, encompassing the number of accessible active sites and the efficiency of interfacial water supply and H_2_ removal. The ECSA‐normalized LSV curves further confirm the superior intrinsic activity of the Ni‐PTFE/NM electrode (Figure S13; Figure 2b), which generates higher current densities than other electrodes at identical potentials. We compared the HER activity of electrodes prepared with different PTFE loadings and conducted relevant control experiments to ensure that the improvement was not caused by CTAB (Figure S14a,b). The Tafel slope of the Ni‐PTFE/NM electrode derived from the LSV curves further suggests more rapid HER kinetics for the Ni‐PTFE/NM electrode, with a value of 30 mV·dec^−1^, approximately half that of the Ni/NM electrode (Figure 2c). Additionally, the normalized Tafel slopes indicate that the NM and Ni/NM electrodes exhibit similar slopes, while the Ni‐PTFE/NM electrode shows a relatively lower slope, suggesting that the improvement in the Tafel slope is not attributed to structural changes (Figure S14c). Nyquist plots obtained via electrochemical impedance spectroscopy (EIS) were fitted using a single‐time‐constant model (Figure 2d) [47, 48]. The fitting results (Table S2) demonstrate that the Ni‐PTFE/NM electrode exhibits the lowest charge‐transfer resistance, suggesting that the introduction of PTFE effectively facilitates electron‐transfer processes. This observation aligns well with the improved HER kinetics derived from the Tafel analysis. The electrode exhibits performance comparable to/superior to the state‐of‐the‐art non‐precious metal electrodes reported in 2025 (Figure 2e; Table S3).

### 2.3 Theoretical Model of Hydrophilic–Hydrophobic Structure

To gain deeper insights into the microscopic mechanisms, DFT calculations were performed to investigate how the hydrophilic–hydrophobic structure regulates HER kinetics at the electrode interface. A comparative study was conducted on two model catalytic layers: Ni and Ni‐PTFE. Figure S15 illustrates the atomic configurations of the two catalytic layers. By examining the distribution of interfacial water on both catalytic layers, we observed that the surface water distribution is relatively uniform on the Ni surface (Figure 2f). In contrast, after introducing hydrophobic PTFE, the interfacial water on the Ni‐PTFE surface is rearranged into a wave‐like stratified distribution centered around the PTFE segments (Figure 2g), in which alternating water‐rich and water‐deficient regions emerge. This wave‐like stratification creates spatially distinct local microenvironments on the Ni‐PTFE surface, potentially enabling the differentiation of active sites with different catalytic functions. To quantitatively evaluate how the local microenvironment affects adsorption behavior, four representative Ni surface sites were selected from the water‐rich and water‐deficient regions (Figure S15a), and the Ni site at 0 was used as the reference point (Figure S15b). Due to the inherent fluidity of water, we employed ab initio molecular dynamics (AIMD) simulations to capture the dynamic evolution of the energy barriers (Δ*E*) for water dissociation forming *H (*denotes active sites) across various active sites, thereby providing a more objective representation of realistic conditions. The results show that the Δ*E* on the Ni surface is 0.90 eV (Figure 2h). In contrast, as shown in Figure 2i and Figure S16, the Δ*E* values at representative sites in the water‐rich region are 0.72 eV (site 1) and 0.49 eV (site 3), whereas in the water‐deficient region, the Δ*E* values increase to 1.40 eV (site 2) and 1.17 eV (site 4). These results indicate that the water‐rich regions exhibit significantly lower Δ*E*, suggesting more favorable thermodynamics for HER, while the water‐deficient regions likely facilitate H_2_ removal.

**FIGURE 3:**
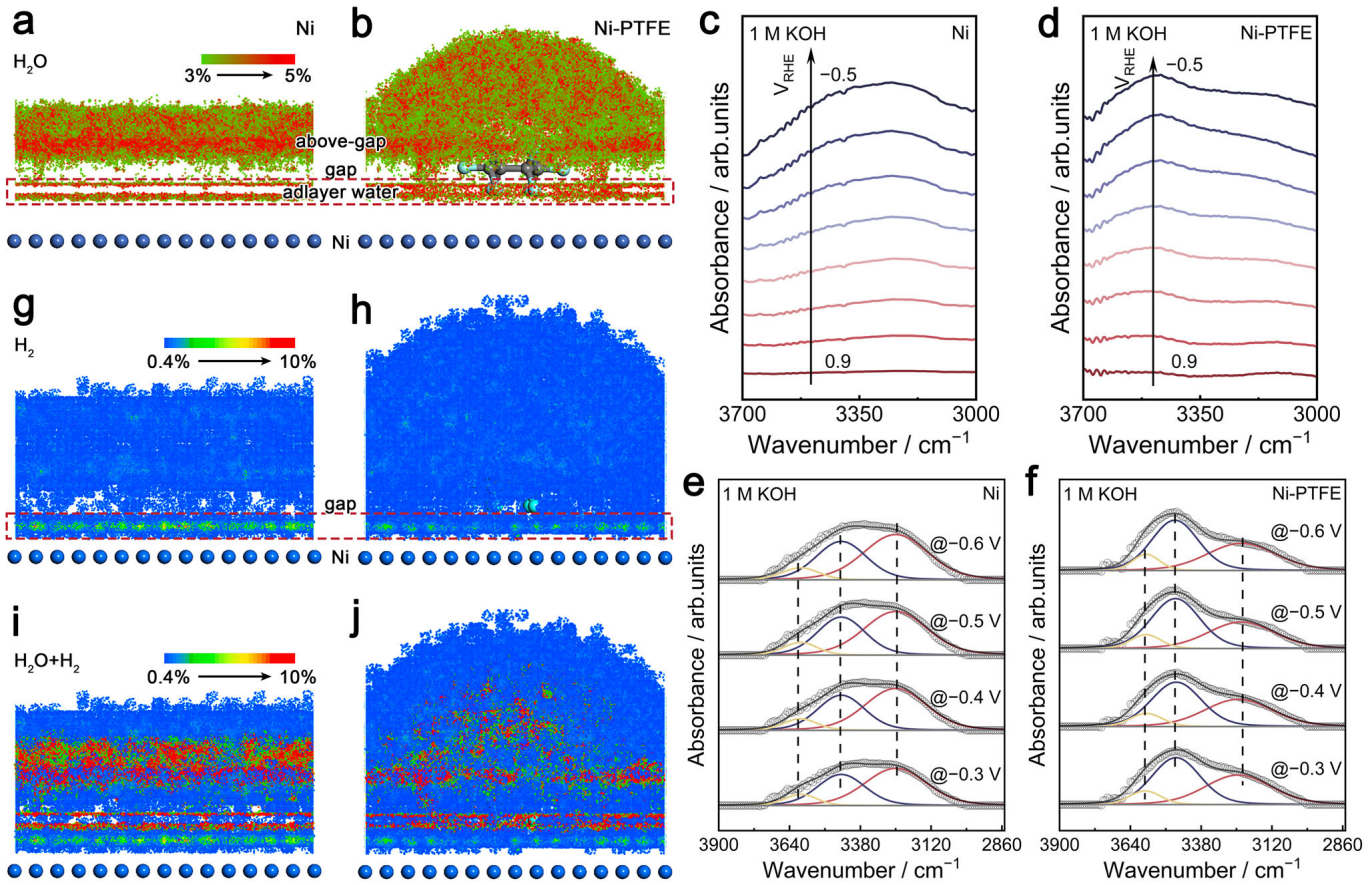
Interfacial water structure and hydrogen distribution modulated by hydrophobic PTFE. Front view of the water molecular number density distribution at the interface of (a) Ni and (b) Ni‐PTFE. In situ ATR‐SEIRAS spectra recorded during the I‐t curve of (c) Ni and (d) Ni‐PTFE electrodes at potential from 0.9 to −0.5 V versus RHE in 1 M KOH. Deconvoluted O─H stretching bands from in situ ATR‐SEIRAS spectra of (e) Ni and (f) Ni‐PTFE electrodes at potentials from −0.3 to −0.6 V versus RHE in 1 M KOH. Front view of the hydrogen molecule number density distribution at the interface of (g) Ni and (h) Ni‐PTFE. Front view of the hydrogen molecule and water molecule number density distribution at the interface of (i) Ni and (j) Ni‐PTFE.

A lower Δ*E* corresponds to a reduced energy barrier for water dissociation and hydrogen adsorption, thereby facilitating HER kinetics. The Δ*E* values at the water‐rich region are significantly lower than those on pure Ni, consistent with the enhanced electrochemical activity observed experimentally. To elucidate the origin of these differences, we further analyzed the interfacial water distribution characteristics on both catalytic layers using molecular simulations. Figure S17 shows the 3D spatial distribution of H_2_O molecules near the catalyst layer for Ni and Ni‐PTFE. A dense water layer (adlayer water) forms adjacent to the Ni surface of both catalysts, consistent with the hydrophilic nature of Ni. However, a prominent interfacial gap zone typically forms above the adlayer water, where the water concentration decreases abruptly. This gap zone has been identified as a critical factor limiting HER kinetics [2, 49]. As shown in Figure S17b, the introduction of PTFE disrupts the continuity of the gap zone. We further analyzed the interfacial water distribution using molecular number density profiles. As shown in Figure 3a and Figure S18a, water adjacent to the Ni surface can be divided into bulk‐like and adlayer water, with pronounced gap zones separating successive layers. The water distribution on both sides of the gap zone is relatively uniform, and water must traverse the gap to reach Ni active sites for the catalytic reaction. Under these conditions, Δ*E* across different Ni active sites remains relatively uniform (0.90 eV). However, on the Ni‐PTFE surface, hydrophobic PTFE domains intrude into the otherwise hydrophilic Ni matrix. This interfacial intrusion disrupts the natural structural hierarchy of the electrode–electrolyte interface, particularly the sequential arrangement of the adlayer water and the adjacent gap zone (Figure 3b; Figure S18b). As a result, water molecules rearrange near the PTFE, forming vertically extended water channels in the water‐rich regions. These water channels continuously transport bulk water to the near‐interface region of active sites, significantly enhancing the local water activity within the gap zone. Since the HER rate‐determining step in alkaline media is typically water dissociation (Volmer step: H_2_O + * + e^−^ → H* + OH^−^) [50, 51, 52], the continuous replenishment of water at metal active sites effectively lowers the dissociation barrier. DFT results confirm that at the water‐rich region sites in the Ni‐PTFE system, Δ*E* decreases from 0.90 to 0.49–0.72 eV.

To verify the water channels induced by interfacial water rearrangement, we employed in situ ATR‐SEIRAS to probe interfacial water. Figure 3c,d shows the in situ ATR‐SEIRAS spectra of interfacial water on Ni and Ni‐PTFE in 1 M KOH at different potentials. During HER, water accumulation at the electrode surface can be observed on both Ni and Ni‐PTFE due to the applied electric field. According to a previous study [2], the O─H stretching band of H_2_O can be deconvoluted into three peaks: adlayer water at ∼3250 cm^−1^, H_2_O_(above‐gap)_ at ∼3450 cm^−1^, and H_2_O_(gap)_ at ∼3610 cm^−1^. Based on the deconvolution fractions of the O─H band obtained at different potentials on Ni and Ni‐PTFE (Figure 3e,f; Table S4), the interfacial fraction of H_2_O_(above‐gap)_ on Ni‐PTFE is significantly higher than that on Ni. This increase occurs at the expense of the adlayer water fraction, implying that the hydrophobic PTFE disrupts the adlayer water layer, resulting in alternating periodic variations of water‐deficient and water‐rich regions. In alkaline HER, where H_2_O is the reactant, a less rigid H‐bond network lowers intermolecular attraction, promoting the migration of water molecules toward active sites and thereby reducing the Volmer step barrier [3, 53, 54]. On the other hand, the PTFE‐induced reconstruction of the interfacial H‐bond network promotes the formation of water channels, enhancing the exchangeability of water molecules on both sides of the gap. This is specifically reflected in the increased proportion of H_2_O_(gap)_, which correlates with the interfacial water rearrangement and the reduction in ΔE discussed above. Furthermore, Figure S19a,b shows the in situ ATR‐SEIRAS spectra for electrodes with different PTFE coverages. At low PTFE loading, the spectrum of Ni‐0.1PTFE closely resembles that of bare Ni, indicating that PTFE has a negligible influence on interfacial water distribution at this coverage (Figure S19c; Table S4). In contrast, under excessive PTFE loading, Ni‐10PTFE exhibits a significantly increased proportion of H_2_O_(above‐gap)_, a slightly decreased proportion of adlayer water, and a reduction in H_2_O_(gap)_ compared to Ni (Figure S19d; Table S4). TEM images reveal significant local aggregation and stacking of PTFE (Figure S9f–h). This likely indicates that the agglomerated PTFE not only covers a large number of active sites, reducing the proportion of adlayer water, but also hinders water exchange between the two sides of the gap.

The density of H_2_ near active sites affects not only the reaction rate but also the thermodynamic equilibrium. As shown in Figure 3g,h, H_2_ in the region adjacent to the catalytic layer forms quasi‐layered, clustered structures. On Ni‐PTFE, compared with Ni, the H_2_ density near active sites is significantly lower, while the interlayer exchange frequency of H_2_ is higher. Figure 3i,j show the molecular number density profiles of H_2_ and water molecules at the interface. The H_2_ molecule layer is closer to the catalyst surface than the water molecule layer, potentially leading to a higher H_2_ desorption energy barrier. In contrast to the Ni catalyst layer, the Ni‐PTFE system, due to the presence of a water‐deficient region, reduces the H_2_ desorption energy barrier near the interface, thereby lowering the H_2_ molecule density at active sites. These findings indicate that the Ni‐PTFE system exhibits distinct molecular distribution characteristics, which may reduce the adsorption/desorption energy barriers and influence the thermodynamic behavior and overall reaction rate of the catalytic process.

### 2.4 Wettability Characterization

**FIGURE 4:**
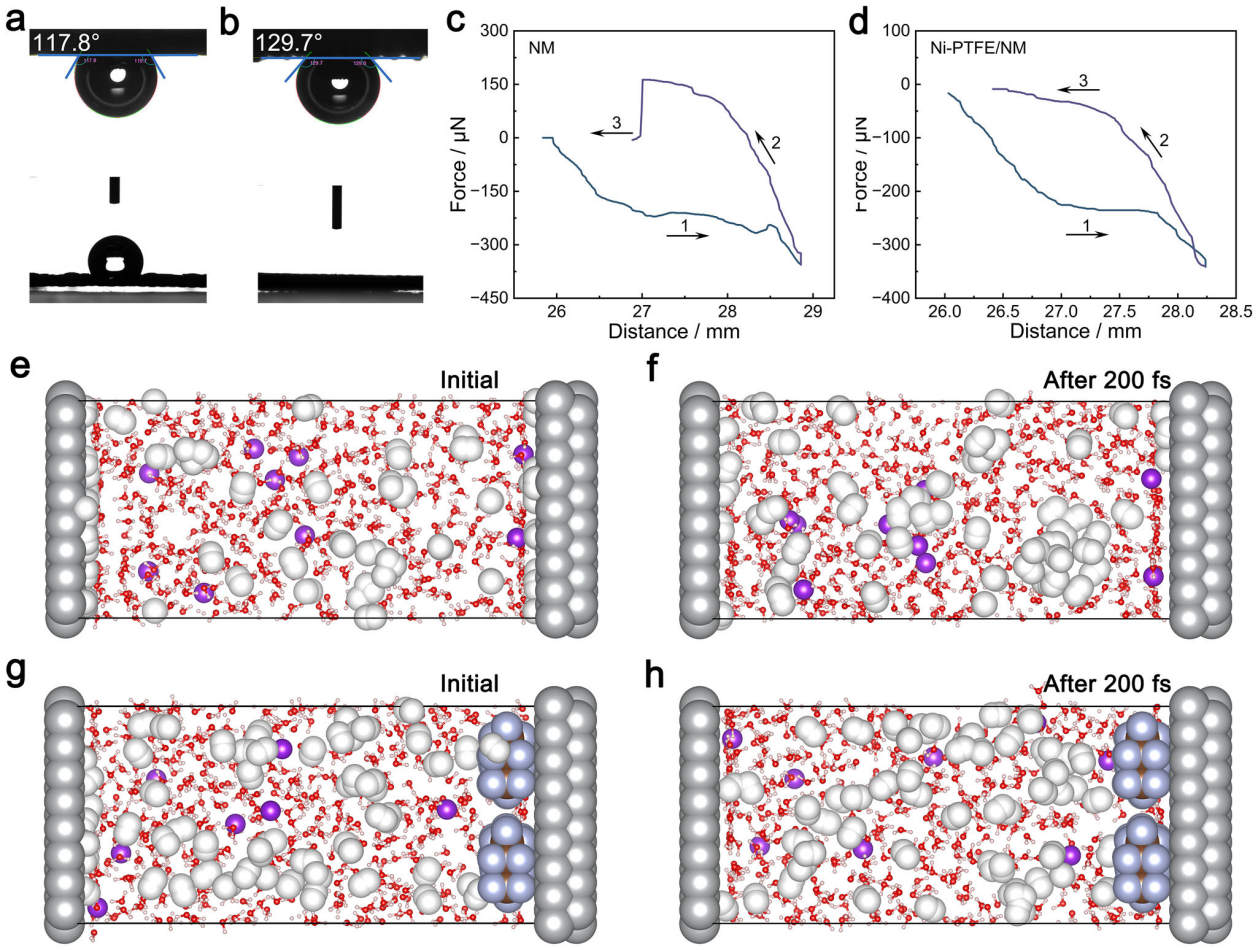
Wettability and MD simulations of H_2_ detachment on Ni‐PTFE. Wetting tests and underwater bubble CA tests on electrodes of (a) NM and (b) Ni‐PTFE/NM electrodes. Adhesive forces measurements of the gas bubble on (c) NM and (d) Ni‐PTFE/NM electrodes. Snapshots of the MD simulated interfacial structure on the Ni at (e) the initial state and (f) the 200 fs state. Representative snapshots of the MD simulated interfacial structure on the Ni‐PTFE at (g) the initial state and (h) the 200 fs state.

**FIGURE 5:**
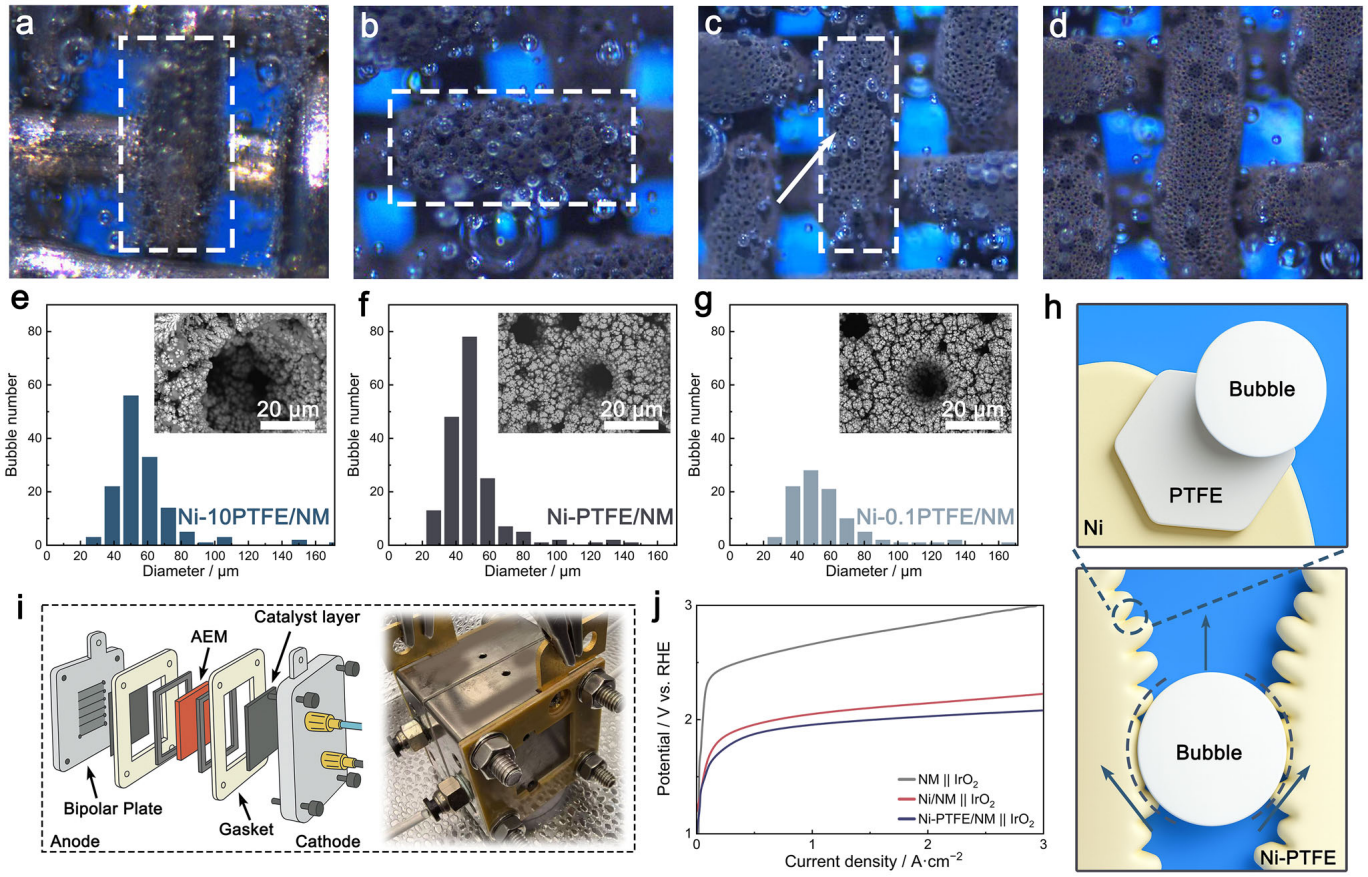
Bubble behavior and gas channel formation on Ni‐PTFE/NM electrode during HER. Optical images of bubbles on the surface of electrodes: (a) NM, (b) Ni‐10PTFE/NM, (c) Ni‐PTFE/NM, and (d) Ni‐0.1PTFE/NM at 10 mA·cm^−2^. Bubble size and number distribution on (e) Ni‐10PTFE/NM, (f) Ni‐PTFE/NM and (g) Ni‐0.1PTFE/NM electrodes during HER. (h) Schematic illustration of gas channels facilitating bubble detachment. (i) The structural schematic diagram and operational photograph of AEMWE. (j) Polarization curves of AEMWEs with different electrodes in 1 M KOH at room temperature.

The above experimental and theoretical studies demonstrate the superiority of the hydrophilic‐hydrophobic structural design. However, a critical question remains: can this hydrophilic‐hydrophobic structure enhance catalytic performance under realistic operating conditions? During HER, gas bubbles accumulate at the electrocatalyst–electrolyte interface, hindering contact between active sites and electrolyte while introducing additional impedance that limits electrolysis efficiency [27, 31, 36, 55]. To explore this question, we investigated the bubble dynamics of the Ni‐PTFE/NM electrode (Figures 4 and 5).

Wettability tests indicate that the pristine NM electrode exhibits poor aqueous electrolyte infiltration (Figure 4a **lower**) while bubble measurement shows a contact angle (CA) of 117.8° (Figure 4a **upper**). With the introduction of micro/nanoscale architectures, the electrode's surface properties undergo pronounced transitions. In particular, the Ni/NM electrode is rapidly wetted by the electrolyte within microseconds, and its bubble CA increases to 138.4° (Figures S20a–c and S21a). After doping with PTFE, the Ni‐PTFE/NM electrode maintains excellent electrolyte permeability, although the bubble CA decreases proportionally with the PTFE content (Figure 4b; Figure S20d–i and S21b,c). Adhesion force measurements provide further insight into wettability: the NM electrode demonstrates a bubble adhesion force of 150 µN (Figure 4c), whereas the Ni/NM and Ni‐PTFE/NM electrodes exhibit negligible adhesive strength (Figure 4d; Figure S22), indicating that trace amounts of PTFE do not significantly alter the electrode's wetting behavior.

The introduction of PTFE significantly impacts the nucleation and transfer of H_2_ bubbles. As verified by SEM and TEM, the PTFE introduced via electrodeposition exhibits relatively small particle dimensions. It is well established that hydrophobic surfaces foster enhanced gas contact in aqueous environments [37, 56]. To gain insights into these phenomena at the molecular scale, MD simulations are employed to probe the diffusion behavior at the interface. Comparative models of a pure Ni catalytic surface and a Ni surface modified with PTFE reveal distinct alterations in the adsorption, transport, and aggregation of H_2_ molecules. As illustrated in Figure 4e, numerous H_2_ molecules accumulate near the Ni catalytic surface. Even after 200 fs, significant H_2_ aggregation remains adjacent to the Ni surface. This thermodynamically increases the difficulty of product formation and hinders the adsorption–desorption processes at active sites (Figure 4f). In contrast, PTFE introduction into the Ni catalyst significantly alters the desorption and transfer pathways of H_2_ molecules, guiding their gradual migration toward PTFE regions (Figure 4g,h) and ultimately leading to their accumulation near the PTFE (Figure S23). Due to the limited miscibility of PTFE with the surrounding aqueous phase, a localized hydrophobic region is formed. This hydrophobic interface provides a favorable environment for the aggregation of H_2_ molecules and facilitates bubble nucleation and growth. Notably, the accumulation of H_2_ on the PTFE surface is primarily governed by hydrophobic interactions rather than by specific adsorption [57]. Since PTFE does not form stable chemical bonds with H_2_, the molecules remain relatively unbound on its surface, enabling ready bubble detachment once formed. Meanwhile, because the Ni catalytic layer is not excessively occupied by bubbles, it can continue to facilitate water dissociation and H_2_ molecules generation, thereby supporting ongoing reactions.

### 2.5 Bubble Growth and Detachment

By employing bubble‐templating electrodeposition, a porous electrode with interconnected microscale gas channels was fabricated. Such a micro/nanostructured surface not only markedly enhances electrolyte hydrophilicity but also provides efficient pathways for bubble nucleation and rapid detachment. High‐speed imaging indicates that, compared to the planar NM substrate (Figure 5a), the Ni‐PTFE/NM electrode enable continuous high‐throughput bubble escape via these gas channels (Figure 5b–d), resulting in notably improved diffusion efficiency. Interestingly, bubble size closely aligns with the gas channel diameter, largely owing to capillary effects [58, 59]. Since PTFE loading modifies the dimensions of the gas transport channels, we systematically investigated this effect (Table S1). When the channel diameter is relatively large, the ample interior space weakens capillary forces during the early growth stage, allowing bubbles to grow to larger sizes within the channels (Figure 5b). In contrast, smaller channel diameters with more densely distributed pores exert strong capillary pressure, which pushes nascent bubbles out in earlier growth stages, leading to smaller bubbles upon detachment (Figure 5c). However, if the channels are excessively narrow, limited nucleation and growth sites reduce bubble generation (Figure 5d). A statistical assessment of bubble sizes (Figure 5e–g) verifies that the Ni‐PTFE/NM electrode yields more bubbles with smaller sizes [60]. Consequently, an optimally sized gas channel is advantageous for promoting the rapid detachment of smaller bubbles and enhancing overall catalytic performance. The schematic illustration in Figure 5h depicts the detachment process of hydrogen bubbles on the Ni‐PTFE catalyst layer. As previously analyzed, H_2_ molecules tend to accumulate on the PTFE surface during electrocatalysis, and PTFE facilitates efficient H_2_ nucleation. With continued reaction, these initial nucleation sites give rise to growing H_2_ bubbles. Once the bubbles reach a critical size, the gas channels on the electrode surface provide significant capillary forces that drive the rapid detachment of bubbles from the catalytic interface while they remain relatively small. Therefore, the combined effect of PTFE modification and gas channel architecture effectively alters the H_2_ nucleation and diffusion pathways, enabling more orderly H_2_ bubble generation and detachment.

Additionally, we performed slope calculations in the diffusion‐controlled region of the LSV curves (Figure 2a), which revealed that the Ni‐PTFE/NM electrode exhibited the smallest slope, indicating its superior mass transport performance (Figure S24). Furthermore, high‐speed imaging was employed to investigate the bubble detachment behavior of the Ni‐PTFE/NM electrode at a current density of 500 mA·cm^−2^ (Figure S25). Although the direct detachment of bubbles on the electrode surface was not directly observed (Figure S25a), upon ceasing the applied voltage and reducing the overall bubble population, a significant number of bubbles were clearly observed escaping from the gas channels (Figure S25b). This observation demonstrates that the gas channels maintained their critical role in reducing bubble size even under high current densities. Since the nucleation, growth, and detachment of bubbles on the electrode surface significantly influence potential fluctuations during constant current testing [27, 60], we further examined the performance of different electrodes at a current density of 500 mA·cm^−2^. The results showed that electrodes with micro‐nanostructures exhibited relatively smaller potential fluctuations during operation, whereas the NM electrode displayed pronounced potential oscillations (Figure S26a). The amplitude and frequency of potential oscillations are closely associated with the size and quantity of detached bubbles. Analysis revealed that the NM electrode exhibited a potential oscillation amplitude as high as 18 mV (Figure S26b), whereas the Ni‐PTFE/NM electrode showed a significantly smaller oscillation amplitude of only 5 mV, which was also lower than those of the Ni‐0.1PTFE/NM and Ni‐10PTFE/NM electrodes (Figure S26c,d). This result further confirms that the bubbles on the surface of the Ni‐PTFE/NM electrode detach at smaller sizes. In addition, the oscillation frequency of the Ni‐PTFE/NM electrode was significantly different from that of the NM electrode, providing further evidence that bubbles detach more frequently from the surface of the Ni‐PTFE/NM electrode. The high‐frequency detachment of small‐sized bubbles effectively reduces the coverage of active sites and the bubble‐induced resistance at high current densities. This results in significantly improved water‐splitting efficiency while minimizing additional energy loss for the Ni‐PTFE/NM electrode.

To comprehensively evaluate the electrochemical stability of the Ni‐PTFE/NM electrode, a continuous 50‐h chronopotentiometry (CP) test was conducted. As depicted in Figure S27a, the Ni‐PTFE/NM electrode maintained stable catalytic performance without a significant increase in the required potential. Following the CP test, a subsequent LSV measurement revealed that the overpotential for HER at a current density of 50 mA·cm^−2^ increased by only 11 mV, indicating that the electrode retained its high catalytic activity after long‐term testing (Figure S27b). SEM analysis was performed on the Ni‐PTFE/NM electrode post‐reaction, and the images in Figure S28 demonstrated that the surface structure remained largely intact after electrolysis. This suggests that the gas channels effectively facilitated continuous and efficient gas detachment, mitigating mechanical damage caused by the detachment of large gas bubbles. Moreover, elemental mapping confirmed that the PTFE component remained uniformly distributed without detectable loss or migration, further supporting the chemical stability of the composite in the catalytic environment (Figure S29). Finally, XRD analysis was performed to examine the crystallographic structure of the Ni‐PTFE/NM electrode before and after testing (Figure S30). The results showed that both the position and intensity of the diffraction peaks remained nearly unchanged, indicating that the crystal structure of the electrode was well preserved during extended electrochemical operation. Furthermore, full‐cell performance of the Ni‐PTFE/NM electrode was evaluated via an anion‐exchange membrane water electrolyzer (AEMWE) for full‐cell performance evaluation using a 1 cm^2^ geometric area (Figure 5i). IrO_2_‐coated titanium felt was employed as the anode. The Ni‐PTFE/NM || IrO_2_ configuration exhibited the lowest cell voltage among the tested electrodes, requiring only 1.91 and 2.0 V to achieve current densities of 1 and 3 A cm^−2^, respectively (Figure 5j). As shown in Figure S31, the Ni‐PTFE/NM || IrO_2_ cell demonstrated the lowest slope, indicating superior mass transport capability [61]. This performance enhancement can be attributed not only to the hydrophilic‐hydrophobic heterostructure of Ni‐PTFE, which reduces the HER energy barrier and accelerates reaction kinetics, but also to the improved bubble adsorption‐detachment behavior. Specifically, the distinct surface regions for catalysis (Ni) and bubble nucleation (PTFE) optimize gas management and active site availability, thereby facilitating electrolyte penetration and bubble release.

## 3 Conclusion

In summary, this study demonstrates that incorporating hydrophobic PTFE onto the Ni catalyst layer effectively modulates the interfacial water structure, thereby enhancing HER performance. The Ni‐PTFE composite achieves a notable reduction in HER overpotential, from 36 to 19 mV at 10 mA·cm^−2^. In situ ATR‐SEIRAS reveals that the fraction of H_2_O_(above‐gap)_ increases from 36.6% to 50.5% at the hydrophilic–hydrophobic Ni‐PTFE interface, promoting proton transfer and water dissociation. Corroborated by DFT calculations, we further illustrate that the hydrophobic PTFE induces a wave‐like rearrangement of interfacial water on the Ni surface, creating alternating water‐rich and water‐deficient regions. This spatial differentiation segregates active sites for water dissociation and H_2_ desorption, thereby optimizing the reaction pathway and accelerating HER kinetics through synergistic improvements in both mass transport and surface reactivity. This work provides not only a strategic approach to designing efficient and stable electrocatalysts through interfacial water regulation, but also offers fundamental insights into the role of hydrophobic modification in electrocatalytic reactions.

## 4 Experimental Section

### 4.1 Materials

Nickel sulfate (NiSO_4_·6H_2_O), boric acid (H_3_BO_3_), ammonium chloride (NH_4_Cl), Polytetrafluoroethylene dispersion (60 wt.%), cetyltrimethylammonium bromide (CTAB), potassium hydroxide (KOH) and hydrochloric acid (HCl) were purchased from China Aladdin Chemical Co. All chemicals were of analytical grade and used without further purification. All experimental solutions were prepared using Milli‐Q ultrapure water (18.2 mΩ·cm) Milli‐Q, Millipore system.

### 4.2 Pretreatment of Nickel Mesh

The nickel mesh with a size of 2 × 3 cm was immersed in a 3 M HCl solution under sonication for 15 min to remove the surface oxides. The treated nickel mesh was washed with ultrapure water and ethanol alternatively, and then dried under vacuum at 60°C.

### 4.3 Electrodeposition of Ni/NM Electrode by Bubble Template Method

A mixture of 0.1 M NiSO_4_, 0.2 M H_3_BO_3,_ and 2 M NH_4_Cl was prepared as plating solution and deposited for 150 s at a current density of 4 A·cm^−2^ using the two‐electrode system. The resulting electrode was designated as Ni/NM.

### 4.4 Electrodeposition of Ni‐PTFE/NM Electrodes

The PTFE dispersion (60 wt.%) and CTAB were added to the plating solution to ensure uniform distribution, with a mass ratio of PTFE to CTAB maintained at 1:1. Deposition was carried out using a two‐electrode system at a current density of 4 A·cm^−2^ for 150s. The resulting electrodes were designated as Ni‐0.1PTFE/NM, Ni‐PTFE/NM, and Ni‐10PTFE/NM.

### 4.5 Electrodeposition of Ni/NM‐PTFE and Ni/NM‐CTAB Electrodes

Only PTFE dispersion (60 wt.%) or CTAB was individually added to the plating solution, and electrodeposition was performed using a two‐electrode system at a current density of 4 A·cm^−2^ for 150s. The resulting electrodes were designated as Ni/NM‐PTFE and Ni/NM‐CTAB.

## Conflicts of Interest

The authors declare no conflicts of interest.

## Supporting information




**Supporting File**: advs75273‐sup‐0001‐SuppMat.docx.

## Data Availability

The data that support the findings of this study are available in the supplementary material of this article.

## References

[1] Y.‐H. Wang , S. Zheng , W.‐M. Yang , et al., “In Situ Raman Spectroscopy Reveals the Structure and Dissociation of Interfacial Water,” Nature 600 (2021): 81–85, 10.1038/s41586-021-04068-z. 34853456

[2] P. Li , Y. Jiang , Y. Hu , et al., “Hydrogen Bond Network Connectivity in the Electric Double Layer Dominates the Kinetic pH Effect in Hydrogen Electrocatalysis on Pt,” Nature Catalysis 5 (2022): 900–911, 10.1038/s41929-022-00846-8.

[3] Y. Huang , Y. Gao , and B. Zhang , “Interfacial Water Regulation for Water‐participating Electrocatalytic Hydrogenation Reactions,” Chemistry (Weinheim An Der Bergstrasse, Germany) 11 (2025): 102533, 10.1016/j.chempr.2025.102533.

[4] I. Ledezma‐Yanez , W. D. Z. Wallace , P. Sebastián‐Pascual , V. Climent , J. M. Feliu , and M. T. M. Koper , “Interfacial Water Reorganization as a pH‐Dependent Descriptor of the Hydrogen Evolution Rate on Platinum Electrodes,” Nature Energy 2 (2017): 17031, 10.1038/nenergy.2017.31.

[5] N. Dubouis , A. Serva , R. Berthin , et al., “Tuning Water Reduction through Controlled Nanoconfinement within an Organic Liquid Matrix,” Nature Catalysis 3 (2020): 656–663, 10.1038/s41929-020-0482-5.

[6] X. You , D. Zhang , X.‐G. Zhang , et al., “Exploring the Cation Regulation Mechanism for Interfacial Water Involved in the Hydrogen Evolution Reaction by In Situ Raman Spectroscopy,” Nano‐Micro Letters 16 (2023): 53, 10.1007/s40820-023-01285-1. 38108934 PMC10728385

[7] A. Montenegro , C. Dutta , M. Mammetkuliev , et al., “Asymmetric Response of Interfacial Water to Applied Electric Fields,” Nature 594 (2021): 62–65, 10.1038/s41586-021-03504-4. 34079138

[8] B. Wu , K. Qi , T. Petit , F. Zhang , Z. J. Xu , and H. Fu , “Modulation of Interfacial Water at Gas–Liquid–Solid Interface for Water Electrolysis,” Angewandte Chemie International Edition 64 (2025): 202507327, 10.1002/anie.202507327. 40905531

[9] S. Li , L. Wu , Q. Liu , et al., “Uncovering the Dominant Role of an Extended Asymmetric Four‐Coordinated Water Network in the Hydrogen Evolution Reaction,” Journal of the American Chemical Society 145 (2023): 26711–26719, 10.1021/jacs.3c08333. 38031299

[10] H. Xie , Z. Zhao , T. Liu , et al., “A Membrane‐based Seawater Electrolyser for Hydrogen Generation,” Nature 612 (2022): 673–678, 10.1038/s41586-022-05379-5. 36450987

[11] H. I. Karunadasa , E. Montalvo , Y. Sun , M. Majda , J. R. Long , and C. J. Chang , “A Molecular MoS _2_ Edge Site Mimic for Catalytic Hydrogen Generation,” Science 335 (2012): 698–702, 10.1126/science.1215868. 22323816

[12] T. Sun , Z. Tang , W. Zang , et al., “Ferromagnetic Single‐Atom Spin Catalyst for Boosting Water Splitting,” Nature Nanotechnology 18 (2023): 763–771, 10.1038/s41565-023-01407-1. 37231143

[13] Z. Chen , N. Han , W. Wei , D. Chu , and B.‐J. Ni , “Dual Doping: An Emerging Strategy to Construct Efficient Metal Catalysts for Water Electrolysis,” EcoEnergy 2 (2024): 114–140, 10.1002/ece2.29.

[14] Y. Zheng , Y. Jiao , A. Vasileff , and S.‐Z. Qiao , “The Hydrogen Evolution Reaction in Alkaline Solution: from Theory, Single Crystal Models, to Practical Electrocatalysts,” Angewandte Chemie International Edition 57 (2018): 7568–7579, 10.1002/anie.201710556. 29194903

[15] Y.‐N. Zhou , W.‐L. Yu , H.‐J. Liu , et al., “Self‐Integration Exactly Constructing Oxygen‐Modified MoNi Alloys for Efficient Hydrogen Evolution,” EcoEnergy 1 (2023): 425–436, 10.1002/ece2.19.

[16] Q. Sha , S. Wang , L. Yan , et al., “10,000‐h‐Stable Intermittent Alkaline Seawater Electrolysis,” Nature 639 (2025): 360–367, 10.1038/s41586-025-08610-1. 40044863

[17] Y. H. Wang , S. Zheng , W. M. Yang , et al., “In Situ Raman Spectroscopy Reveals the Structure and Dissociation of Interfacial Water,” Nature 600 (2021): 81–85, 10.1038/s41586-021-04068-z. 34853456

[18] T. Wang , Y. Zhang , B. Huang , et al., “Enhancing Oxygen Reduction Electrocatalysis by Tuning Interfacial Hydrogen Bonds,” Nature Catalysis 4 (2021): 753–762, 10.1038/s41929-021-00668-0.

[19] M. Wang , K. Sun , W. Mi , et al., “Interfacial Water Activation by Single‐Atom Co–N _3_ Sites Coupled with Encapsulated Co Nanocrystals for Accelerating Electrocatalytic Hydrogen Evolution,” ACS Catalysis 12 (2022): 10771–10780, 10.1021/acscatal.2c02770.

[20] F. Liang , R. van de Krol , and F. F. Abdi , “Assessing Elevated Pressure Impact on Photoelectrochemical Water Splitting via Multiphysics Modeling,” Nature Communications 15 (2024): 4944, 10.1038/s41467-024-49273-2. PMC11164907 38858377

[21] Y. Xiao , C. Tan , F. Zeng , W. Liu , and J. Liu , “Structural Regulation of Amorphous Molybdenum Sulfide by Atomic Palladium Doping for Hydrogen Evolution,” Journal of Colloid and Interface Science 665 (2024): 60–67, 10.1016/j.jcis.2024.03.113. 38513408

[22] A. Angulo , P. van der Linde , H. Gardeniers , M. Modestino , and D. F. Rivas , “Influence of Bubbles on the Energy Conversion Efficiency of Electrochemical Reactors,” Joule 4 (2020): 555–579, 10.1016/j.joule.2020.01.005.

[23] C. Zhang , Z. Xu , N. Han , et al., “Superaerophilic/Superaerophobic Cooperative Electrode for Efficient Hydrogen Evolution Reaction via Enhanced Mass Transfer,” Science Advances 9 (2023): add6978, 10.1126/sciadv.add6978. PMC9848275 36652519

[24] J. Kang , G. Liu , Q. Hu , et al., “Parallel Nanosheet Arrays for Industrial Oxygen Production,” Journal of the American Chemical Society 145 (2023): 25143–25149, 10.1021/jacs.3c05688. 37941374

[25] P. A. Kempler , R. H. Coridan , and L. Luo , “Gas Evolution in Water Electrolysis,” Chemical Reviews 124 (2024): 10964–11007, 10.1021/acs.chemrev.4c00211. 39259040

[26] Z. Zhang , T. Zhao , M. Liu , and L. Jiang , “Superwetting Catalysts: Principle, Design, and Synthesis,” Advanced Materials 37 (2025): 2506058, 10.1002/adma.202506058. 40465368

[27] R. Iwata , L. Zhang , K. L. Wilke , et al., “Bubble Growth and Departure Modes on Wettable/Non‐wettable Porous Foams in Alkaline Water Splitting,” Joule 5 (2021): 887–900, 10.1016/j.joule.2021.02.015.

[28] K. Guan , L. Tao , R. Yang , et al., “Anti‐Corrosion for Reversible Zinc Anode via a Hydrophobic Interface in Aqueous Zinc Batteries,” Advanced Energy Materials 12 (2022): 2103557, 10.1002/aenm.202103557.

[29] Z. Long , C. Yu , M. Cao , J. Ma , and L. Jiang , “Bioinspired Gas Manipulation for Regulating Multiphase Interactions in Electrochemistry,” Advanced Materials 36 (2024): 2312179, 10.1002/adma.202312179. 38388808

[30] L. Zhao , Y. Li , M. Yu , Y. Peng , and F. Ran , “Electrolyte‐Wettability Issues and Challenges of Electrode Materials in Electrochemical Energy Storage, Energy Conversion, and Beyond,” Advanced Science 10 (2023): 2300283, 10.1002/advs.202300283. 37085907 PMC10265108

[31] Z. Lu , W. Zhu , X. Yu , et al., “Ultrahigh Hydrogen Evolution Performance of under‐Water “Superaerophobic” MoS _2_ Nanostructured Electrodes,” Advanced Materials 26 (2014): 2683–2687, 10.1002/adma.201304759. 24488883

[32] S. Mitchell , R. Qin , N. Zheng , and J. Pérez‐Ramírez , “Nanoscale Engineering of Catalytic Materials for Sustainable Technologies,” Nature Nanotechnology 16 (2021): 129–139, 10.1038/s41565-020-00799-8. 33230317

[33] M. Li , C. Liu , Z. Teng , et al., “Manipulating Interfacial Water Configuration via Constructing Asymmetric Structure Unit for Hydrogen Production in Alkaline Seawater,” Advanced Functional Materials 35 (2025): 14517, 10.1002/adfm.202514517.

[34] L. Sun , J. Guo , H. Chen , et al., “Tailoring Materials with Specific Wettability in Biomedical Engineering,” Advanced Science 8 (2021): 2100126, 10.1002/advs.202100126. 34369090 PMC8498887

[35] K. Deng , H. Feng , Y. Zhang , D. Liu , and Q. Li , “Ampere‐level Membrane‐less Water Electrolysis Enabled by Rose‐petal‐effect‐mimetic Interface,” Joule 7 (2023): 1852–1866, 10.1016/j.joule.2023.06.010.

[36] C. Xia , S. Back , S. Ringe , et al., “Confined Local Oxygen Gas Promotes Electrochemical Water Oxidation to Hydrogen Peroxide,” Nature Catalysis 3 (2020): 125–134, 10.1038/s41929-019-0402-8.

[37] D. Wakerley , S. Lamaison , F. Ozanam , et al., “Bio‐Inspired Hydrophobicity Promotes CO_2_ Reduction on a Cu Surface,” Nature Materials 18 (2019): 1222–1227, 10.1038/s41563-019-0445-x. 31384032

[38] D. Iacovetta , J. Tam , and U. Erb , “Synthesis, Structure, and Properties of Superhydrophobic Nickel–PTFE Nanocomposite Coatings Made by Electrodeposition,” Surface and Coatings Technology 279 (2015): 134–141, 10.1016/j.surfcoat.2015.08.022.

[39] K. Helle and F. Walsh , “Electrodeposition of Composite Layers Consisting of Inert Inclusions in a Metal Matrix,” Transactions of the IMF 75 (1997): 53–58, 10.1080/00202967.1997.11871143.

[40] F. Song , W. Li , J. Yang , G. Han , P. Liao , and Y. Sun , “Interfacing Nickel Nitride and Nickel Boosts both Electrocatalytic Hydrogen Evolution and Oxidation Reactions,” Nature Communications 9 (2018): 4531, 10.1038/s41467-018-06728-7. PMC6208398 30382092

[41] F. Kılıç , H. Gül , S. Aslan , A. Alp , and H. Akbulut , “Effect of CTAB Concentration in the Electrolyte on the Tribological Properties of Nanoparticle SiC Reinforced Ni Metal Matrix Composite (MMC) Coatings Produced by Electrodeposition,” Colloids and Surfaces A: Physicochemical and Engineering Aspects 419 (2013): 53–60, 10.1016/j.colsurfa.2012.11.048.

[42] C. Sleigh , A. P. Pijpers , A. Jaspers , B. Coussens , and R. J. Meier , “On the Determination of Atomic Charge via ESCA Including Application to Organometallics,” Journal of Electron Spectroscopy and Related Phenomena 77 (1996): 41–57, 10.1016/0368-2048(95)02392-5.

[43] M. Ackeret , “Polytetrafluoroethylene by XPS,” Surface Science Spectra 1 (1992): 100–103, 10.1116/1.1247678.

[44] J.‐L. Fu , Y. Liu , Y.‐M. Chen , H. Zhang , J.‐P. Qu , and Y.‐B. Kang , “Electrophotocatalysis for Reductive Defluorination of PTFE and PFASs,” Angewandte Chemie International Edition 64 (2025): 202422043, 10.1002/anie.202422043. 40082215

[45] J. Piwowarczyk , R. Jędrzejewski , D. Moszyński , K. Kwiatkowski , A. Niemczyk , and J. Baranowska , “XPS and FTIR Studies of Polytetrafluoroethylene Thin Films Obtained by Physical Methods,” Polymers 11 (2019): 1629, 10.3390/polym11101629. 31600899 PMC6835360

[46] B. Deng , X. He , P. Du , et al., “PTFE as a Multifunctional Binder for High‐Current‐Density Oxygen Evolution,” Advanced Science 11 (2024): 2408544, 10.1002/advs.202408544. 39229933 PMC11538630

[47] B. Wang , M. Lu , D. Chen , et al., “Ni _X_ Fe _y_ N@C Microsheet Arrays on Ni Foam as an Efficient and Durable Electrocatalyst for Electrolytic Splitting of Alkaline Seawater,” Journal of Materials Chemistry A 9 (2021): 13562–13569, 10.1039/D1TA01292D.

[48] G. Li , L. Anderson , Y. Chen , M. Pan , and P.‐Y. Abel Chuang , “New Insights into Evaluating Catalyst Activity and Stability for Oxygen Evolution Reactions in Alkaline media,” Sustainable Energy & Fuels 2 (2018): 237–251, 10.1039/C7SE00337D.

[49] N. Berg , S. Bergwinkl , P. Nuernberger , D. Horinek , and R. M. Gschwind , “Extended Hydrogen Bond Networks for Effective Proton‐Coupled Electron Transfer (PCET) Reactions: the Unexpected Role of Thiophenol and Its Acidic Channel in Photocatalytic Hydroamidations,” Journal of the American Chemical Society 143 (2021): 724–735, 10.1021/jacs.0c08673. 33423466

[50] J. N. Tiwari , M. Umer , G. Bhaskaran , et al., “β–phase Hydroxide‐steered Inner‐hosted Metal Sites for Exceptional Hydrogen Production,” Materials Science and Engineering: R: Reports 168 (2026): 101130, 10.1016/j.mser.2025.101130.

[51] N. K. Oh , J. Seo , S. Lee , et al., “Highly Efficient and Robust Noble‐metal Free Bifunctional Water Electrolysis Catalyst Achieved via Complementary Charge Transfer,” Nature Communications 12 (2021): 4606, 10.1038/s41467-021-24829-8. PMC8322133 34326340

[52] J. Lee , S. J. Lee , S. Lee , and Y.‐K. Han , “Water Dissociation Energy Variations in Hydrogen Evolution Reaction in Transition Metal‐doped MoS_2_ ,” Materials Today Energy 51 (2025): 101911, 10.1016/j.mtener.2025.101911.

[53] X. H. Chen , X. L. Li , T. Li , et al., “Enhancing Neutral Hydrogen Production by Disrupting the Rigid Hydrogen Bond Network on Ru Nanoclusters through Nb _2_ O _5_ ‐Mediated Water Reorientation,” Energy & Environmental Science 17 (2024): 5091–5101, 10.1039/D4EE01855A.

[54] W. Guo , S. Zhang , J. Zhang , et al., “Accelerating Multielectron Reduction at Cu_x_O Nanograins Interfaces with Controlled Local Electric Field,” Nature Communications 14 (2023): 7383, 10.1038/s41467-023-43303-1. PMC10651938 37968299

[55] Y. Li , K. Li , L. Li , et al., “Bubble‐Guidance Breaking Gas Shield for Highly Efficient Overall Water Splitting,” Advanced Materials 36 (2024): 2405493, 10.1002/adma.202405493. 39136062

[56] L. Wang , C. Geng , D. Yu , et al., “Catalytic Performance and Mechanism of PTFE Modified NiCo_2_O_4_ in High‐Salt Organic Wastewater Treatment during Wet Air Oxidation at Ambient Pressure,” Applied Catalysis B: Environmental 334 (2023): 122786, 10.1016/j.apcatb.2023.122786.

[57] J. G. Riess , “Understanding the Fundamentals of Perfluorocarbons and Perfluorocarbon Emulsions Relevant to in Vivo Oxygen Delivery,” Artificial Cells, Blood Substitutes, and Biotechnology 33 (2005): 47–63, 10.1081/BIO-200046659. 15768565

[58] H. S. Rabbani , V. Joekar‐Niasar , and N. Shokri , “Effects of Intermediate Wettability on Entry Capillary Pressure in Angular Pores,” Journal of Colloid and Interface Science 473 (2016): 34–43, 10.1016/j.jcis.2016.03.053. 27042823

[59] B. Dai , K. Li , L. Shi , et al., “Bioinspired Janus Textile with Conical Micropores for Human Body Moisture and Thermal Management,” Advanced Materials 31 (2019): 1904113, 10.1002/adma.201904113. 31456222

[60] Y. Dong , H. Wang , X. Wang , et al., “Superhydrophilic/Superaerophobic NiFe with Internal Bubble Flow Channels for Electrocatalytic Water Splitting,” Chemical Engineering Journal 488 (2024): 150953, 10.1016/j.cej.2024.150953.

[61] H. Wang , X. Xue , M. Fan , et al., “Decoupling Bubble Nucleation from Catalysis to Boost Cu_x_O/NiO Electrocatalytic Water Splitting,” Nano Letters 26 (2026): 1057, 10.1021/acs.nanolett.5c04437. 41525175

