# Supplementary material for: Hydrophobicity Modulated Interfacial Water Distribution for Enhanced Kinetics of HER
Source: Adv Sci (Weinh). 2026 Apr 14;13(39):e75273. doi: 10.1002/advs.75273 (PMC13334965; doi:10.1002/advs.75273)
Supplement: Supplementary file 1 — Supporting File: advs75273‐sup‐0001‐SuppMat.docx. [file ADVS-13-e75273-s001.docx]

Supporting Information

Hydrophobicity Modulated Interfacial Water Distribution for Enhanced Kinetics of HER

Yucheng Dong,^1, 2^ Xinfa Wei,^2^ Xiangdong Xue,^2^ Hanxiao Wang,^2^ Qing Dong^2^ and Jian Liu^1, 2^*

^1^ College of Materials Science and Engineering, Qingdao University of Science and Technology, Qingdao 266042, P. R. China

^2^ State Key Laboratory of Photoelectric Conversion and Utilization of Solar Energy, Qingdao New Energy Shandong Laboratory, Qingdao Institute of Bioenergy and Bioprocess Technology, Chinese Academy of Sciences, Qingdao 266101, P. R. China

^*^ E-mail: liujian@qibebt.ac.cn

1. Physical characterization

The morphology of the newly prepared materials was investigated by field emission scanning electron microscopy (SEM) on a Carl Zeiss Ultra Plus system. Using the CAST 3.0 system, using a 25-gauge pinhole, the contact angle (CA) of the bubble under the electrolyte was measured by the trapped bubble method, and the wetting of the electrolyte was measured by the pendant drop method at 0.8 cm. Adhesion values were measured at Dataphysics DCAT21. The bubble behavior at the electrode was recorded by a high-speed camera (pco.dimax cs) with an electrolysis process performed with Hg/HgO as the reference electrode and graphite rods as the counter electrode, electrolyzed in 1 M KOH. X-ray diffraction (XRD) was performed on a Shimadzu XD-3A instrument equipped with filtered Cu-Kα radiation (λ = 0.15418 nm) and operated at 30 mA and 40 kV. The 2θ scan rate for XRD analysis was set to 5°·min^−1^. A JEOL (JEM-2000 FX) microscope operating at 200 kV was used for transmission electron microscopy (TEM), high-angle annular darkfield scanning transmission electron microscopy (STEM) images. X-ray photoelectron spectroscopy (XPS) was performed on a VG Escalab210 spectrometer with a Mg 300 W X-ray source. To conduct in situ attenuated total reflection surface-enhanced infrared absorption ATR-SEIRAS measurements, a Hg/HgO electrode was used as the reference electrodes in 1 M KOH. A Fourier transform infrared spectrometer equipped with a liquid-nitrogen-cooled mercury cadmium telluride detector was used for the SEIRAS measurements and operated at a resolution of 8 cm^−1^. Unpolarized infrared radiation from an Elema source was focused on the reflection plane with an incident angle of ~60°, and the totally reflected radiation was detected.

2. Electrochemical Characterization

All electrochemical measurements were performed with Hg/HgO as the reference electrode and a graphite rod as the counter electrode. The measured potential was converted to a reversible hydrogen electrode (RHE) using the equation E_RHE_=E_Hg/HgO_+0.059pH+0.098 V. Linear sweep voltammetry (LSV) tests were performed at a scan rate of 5 mV·s^−1^ and all polarization curves were IR compensated (95%). Electrochemical impedance spectroscopy (EIS) was tested in the frequency range from +0.01 Hz to +100 kHz. The electrochemical double layer capacitance (C_dl_) of the samples was determined by cyclic voltammetry (CV) in the Faradaic potential range. Potential-time responses were obtained by chronopotentiometry (CP) at a current density of 50 mA·cm^−2^ for 50 h.

3. Computational details

3.1. Model Construction

The simulation systems were constructed based on a two-layer Ni (111) slab containing 112 Ni atoms. To represent the polytetrafluoroethylene (PTFE) coating in the Ni-PTFE system, a perfluorobutane (C4F10) molecule was introduced as the model compound. An explicit water layer with a thickness of approximately 10 Å was added above the solid surfaces, corresponding to 106 water molecules for the pristine Ni system and 91 water molecules for the Ni-PTFE system. To eliminate artificial interactions between periodic images, a vacuum layer of 20 Å was applied along the z-direction.

3.2. Classical Molecular Dynamics and Monte Carlo Simulations

The interatomic interactions of the entire system, including the Ni slab and PTFE, were described by the Universal Force Field (UFF), which provides broad coverage for both metal and organic elements^[1]^. Atomic charges were assigned using the Charge Equilibration (QEq) method^[2]^. The electrostatic interactions were calculated using the Ewald summation method, while the van der Waals interactions were treated with the atom-based summation method. Prior to the MD production runs, rigorous geometry optimizations were performed to eliminate local structural and stress anomalies, with a strict force convergence criterion of 0.001 kcal/mol/Å. Subsequently, MD simulations were conducted in the *NVT* ensemble at 298 K using the Nosé-Hoover-Langevin (NHL) thermostat^[3]^. The equations of motion were integrated with a time step of 1.0 fs.

To investigate the preferred adsorption configurations, Metropolis Monte Carlo (MC) simulations coupled with a simulated annealing algorithm were carried out^[4]^. The simulated annealing process consisted of 10 cycles, with 100,000 steps per cycle. The force field and charge assignment settings were consistent with those used in the MD simulations.

3.3. Ab Initio Molecular Dynamics (AIMD) Simulations

AIMD simulations were carried out via spin-polarized density functional theory (DFT) within the CP2K package^[5]^. The exchange-correlation effects were treated using the generalized gradient approximation (GGA) with the Perdew-Burke-Ernzerhof (PBE) functional^[6]^, supplemented with Grimme’s DFT-D3 empirical dispersion correction^[7]^. Core electrons were represented by the Goedecker-Teter-Hutter (GTH) pseudopotentials^[8, 9]^, while valence electrons were expanded in a double-ζ valence polarization basis set optimized for molecules and short-range interactions (DZVP-MOLOPT-SR-GTH)^[10]^, which minimizes basis set superposition errors. A plane-wave cutoff of 600 Ry was applied for the auxiliary basis. Geometry optimizations were performed using the Broyden-Fletcher-Goldfarb-Shanno (BFGS) algorithm, with forces converged to within 4.5 × 10^−4^ Hartree/Bohr. The AIMD simulations were performed in the *NVT* ensemble at 298.15 K using the canonical sampling through velocity rescaling (CSVR) thermostat^[11]^ with a time constant of 200 fs. The time step to integrate the Newtonian equations was set to 1.0 fs. The CP2K input files were generated with the assistance of the Multiwfn program^[12, 13]^.


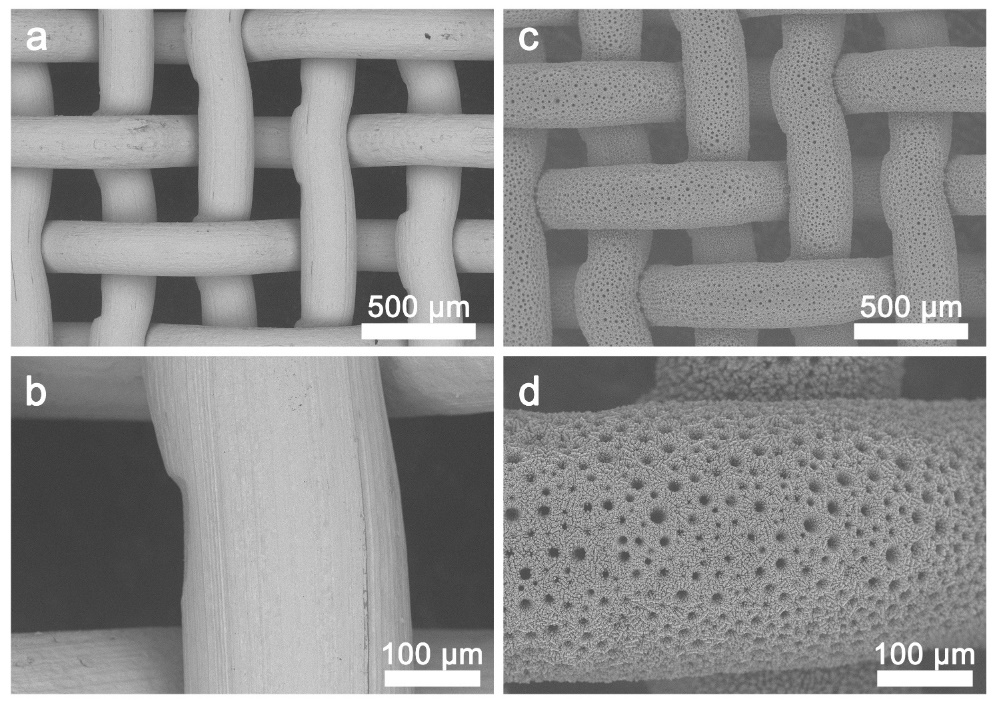


**Figure S1.** SEM images of (a-b) NM and (c-d) Ni/NM electrodes.


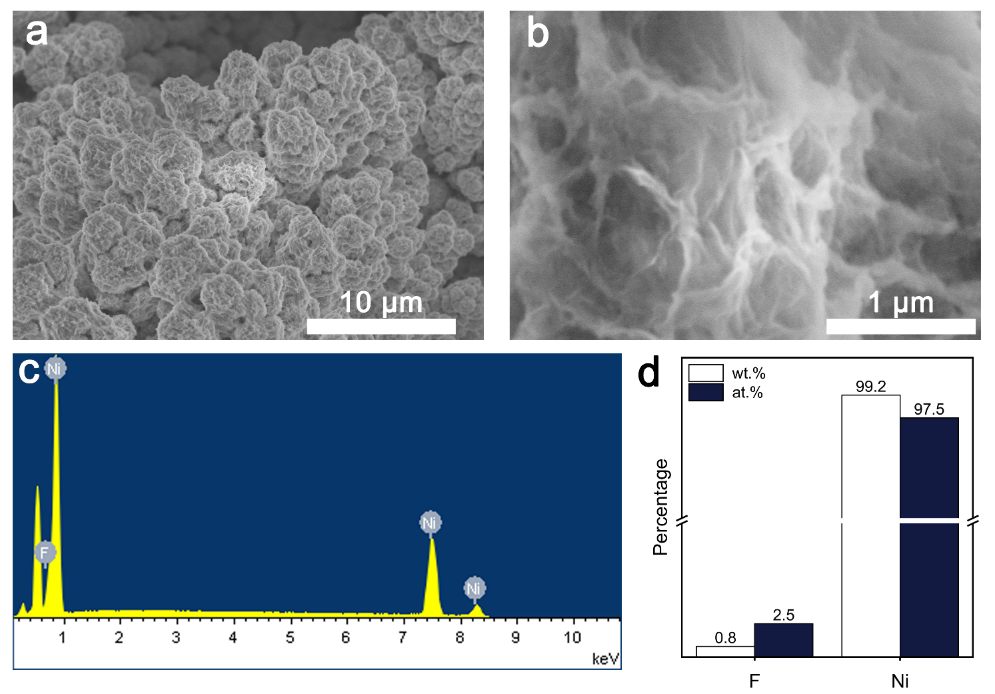


**Figure S2.** SEM images of Ni-PTFE/NM electrode. (a-b) SEM image. (c) EDS spectrum. (d) Elemental composition bar chart.


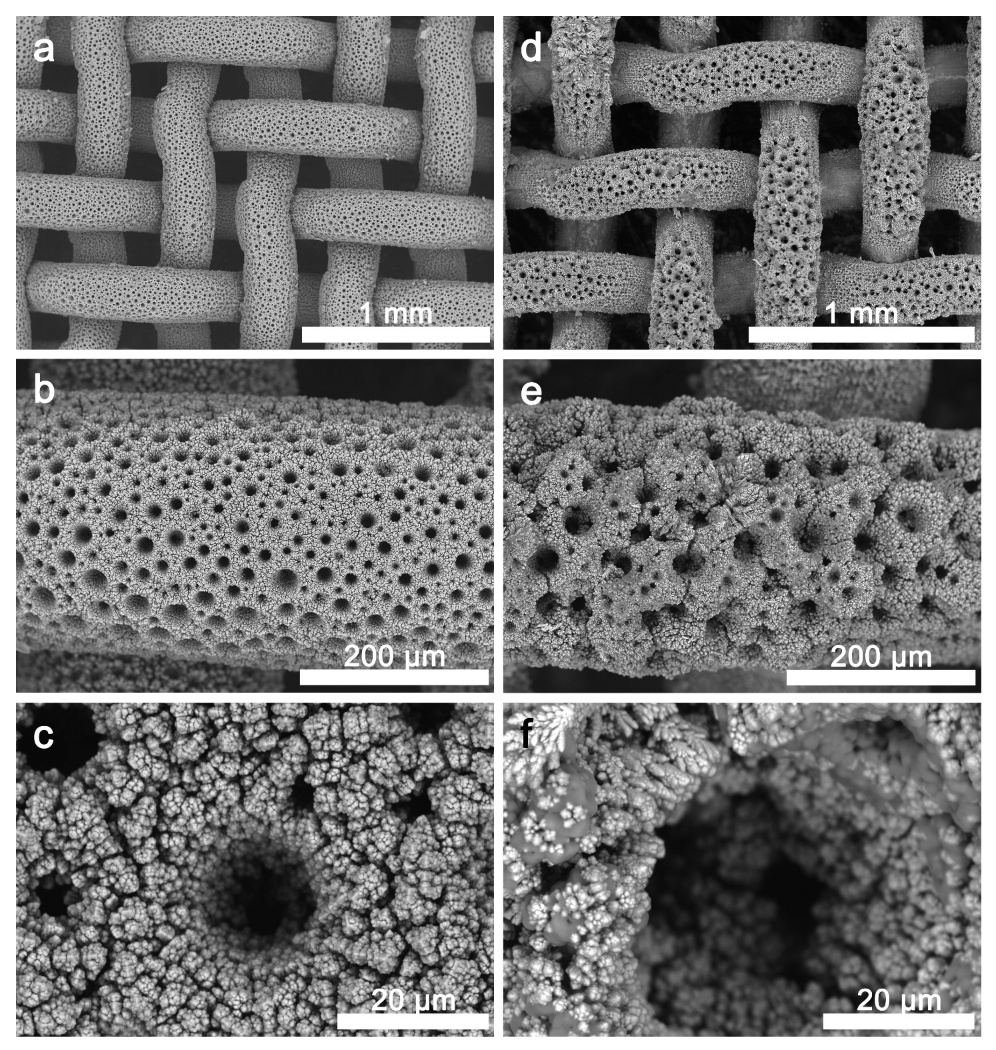


**Figure S3.** SEM images of (a-c) Ni-0.1PTFE/NM and (d-f) Ni-10PTFE/NM electrodes.


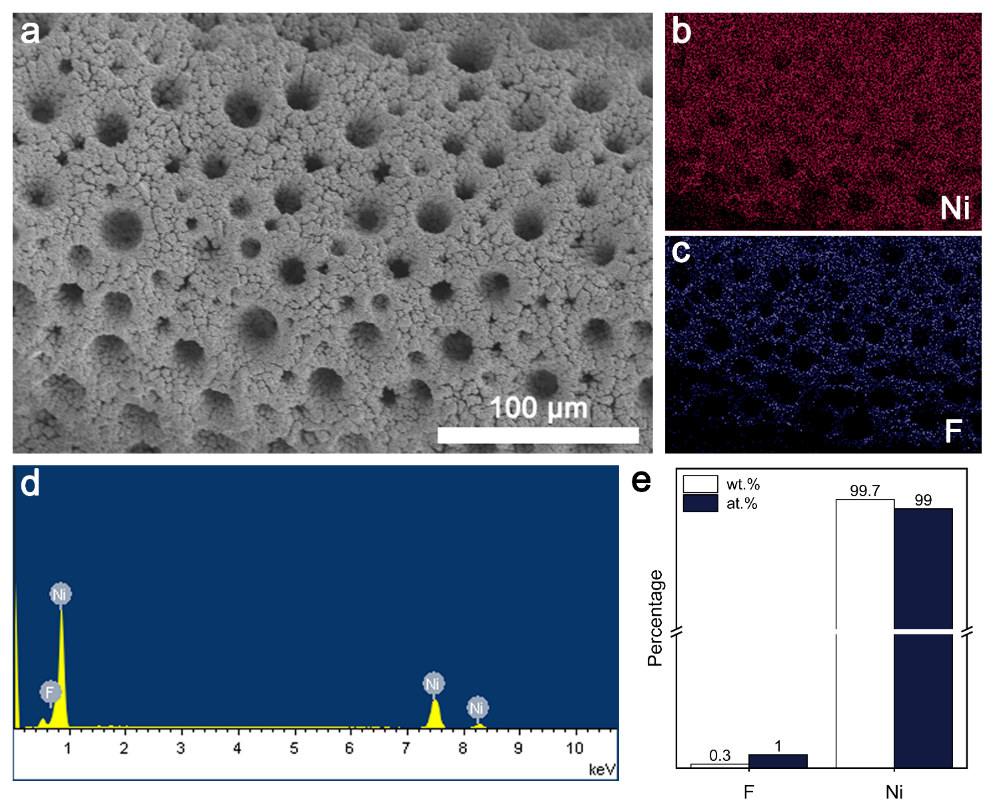


**Figure S4.** SEM images of Ni-0.1PTFE/NM electrode. (a) SEM image. EDS mapping of (b) Ni and (c) F elements. (d) EDS spectrum. (e) Elemental composition bar chart.


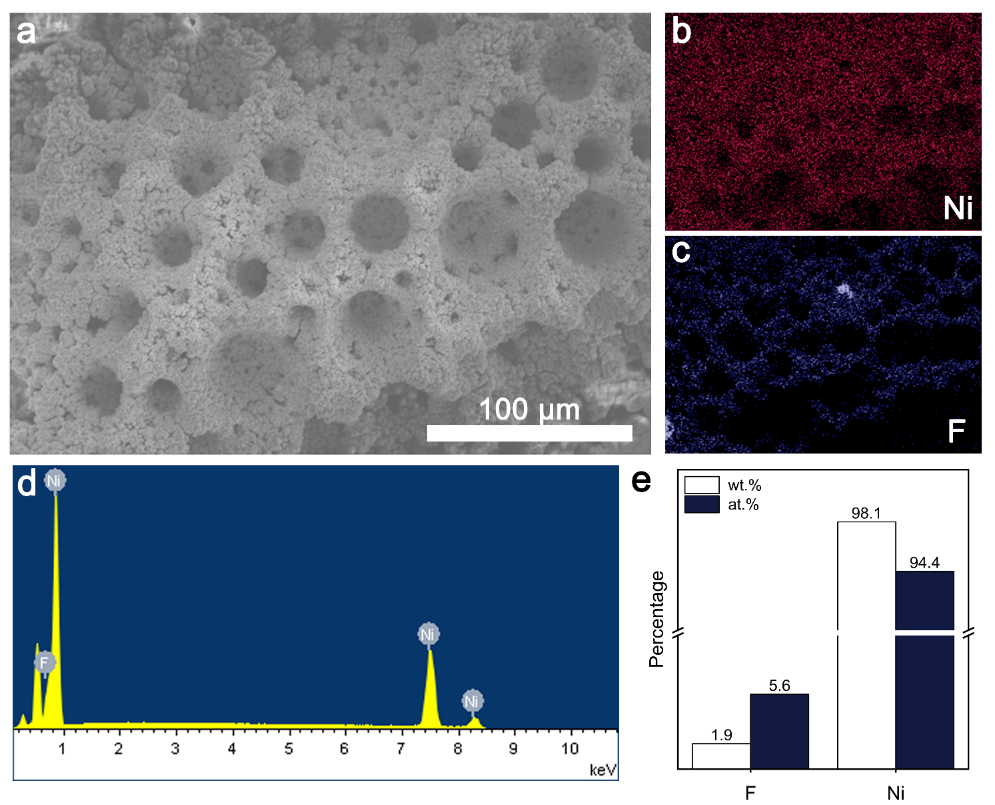


**Figure S5.** SEM images of Ni-10PTFE/NM electrode. (a) SEM image. EDS mapping of (b) Ni and (c) F elements. (d) EDS spectrum. (e) Elemental composition bar chart.


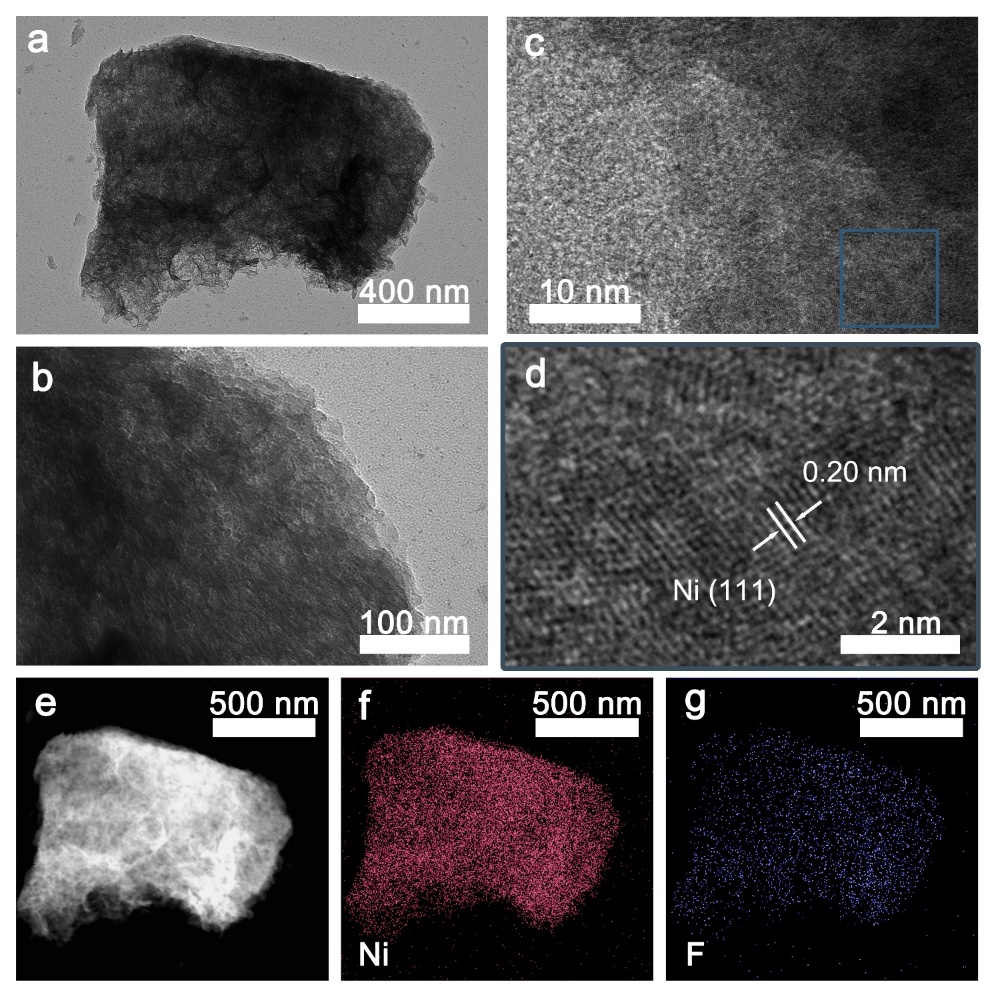


**Figure S6.** TEM of Ni-PTFE/NM electrode. (a-b) TEM image. (c-d) HR-TEM image. (e-g) EDS mapping of Ni and F.


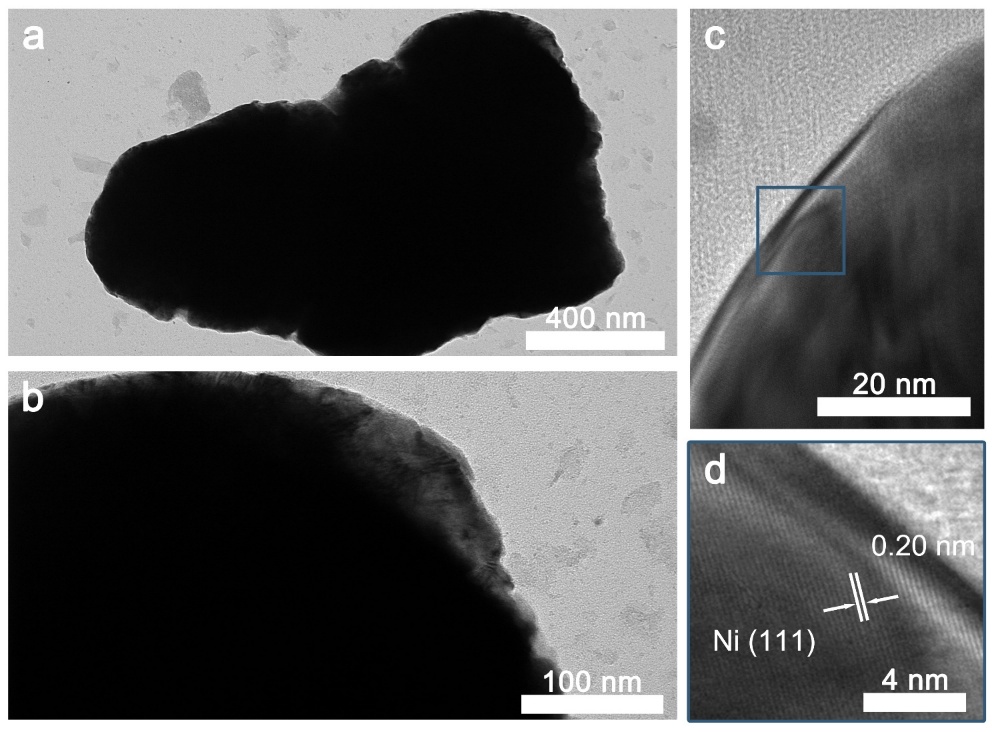


**Figure S7.** TEM of Ni/NM electrode. (a-b) TEM image, (c-d) HR-TEM image.


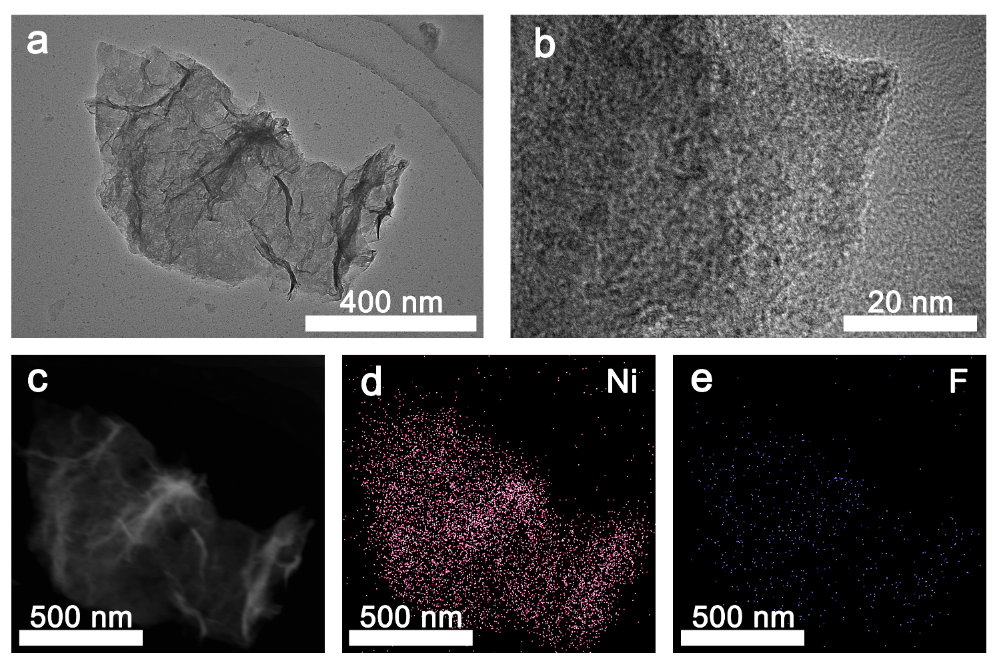


**Figure S8.** TEM of Ni-0.1PTFE/NM electrode. (a) TEM image. (b) HR-TEM image. (c-e) EDS mapping of Ni and F.


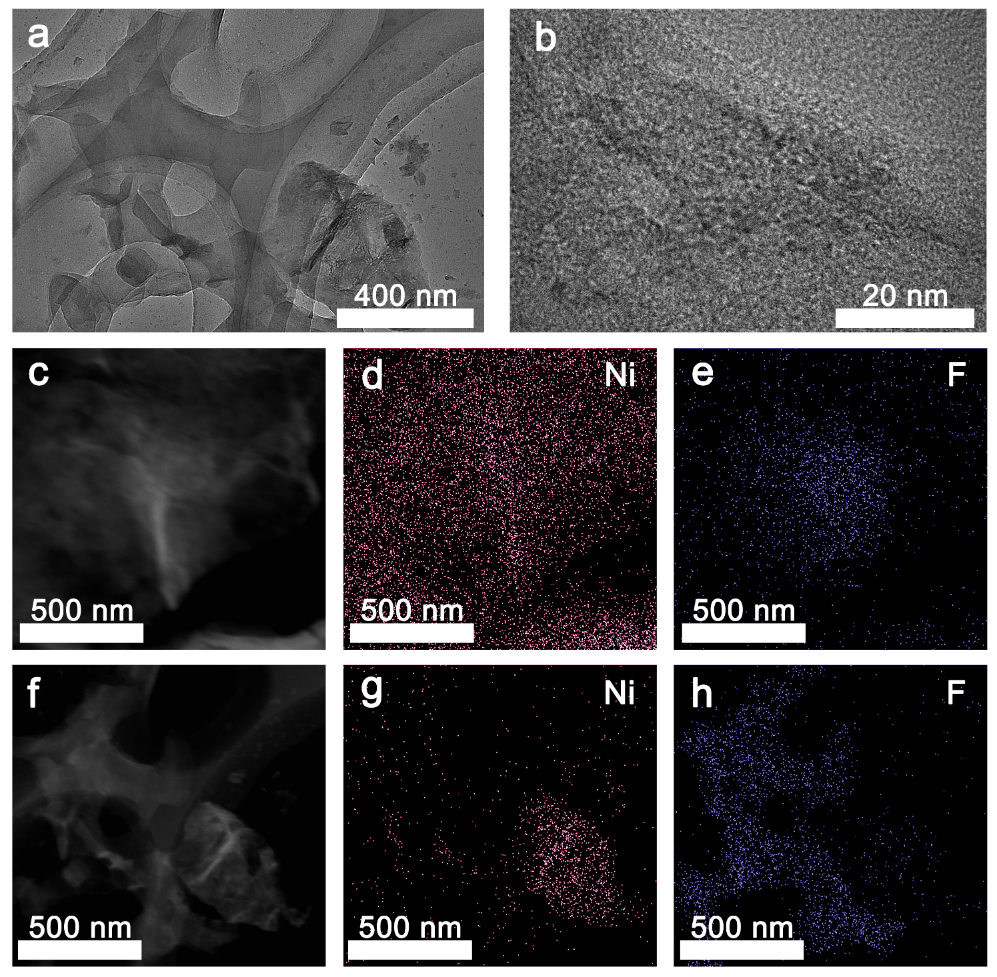


**Figure S9.** TEM of Ni-10PTFE/NM electrode. (a) TEM image. (b) HR-TEM image. (c-h) EDS mapping of Ni and F.


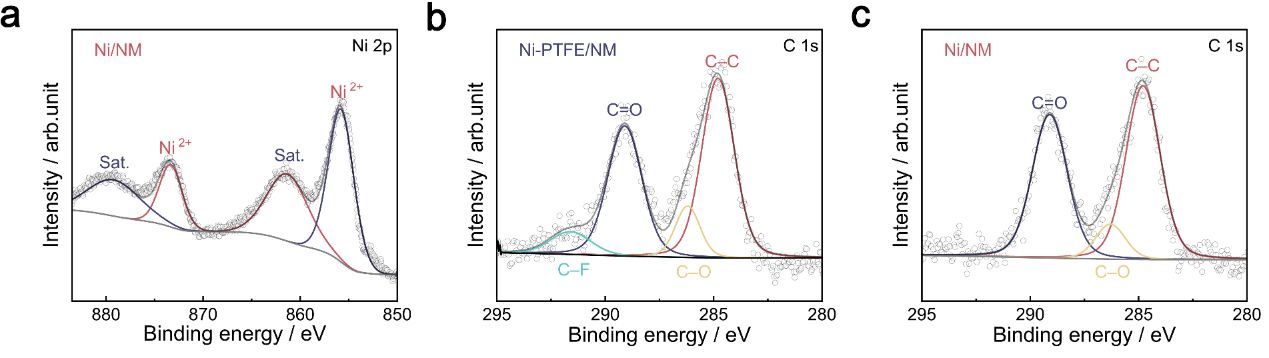


**Figure S10.** High-resolution XPS Ni 2p spectra of the (a) Ni/NM electrode. High-resolution XPS C 1s spectra of the (b) Ni-PTFE/NM and (c) Ni/NM electrodes.


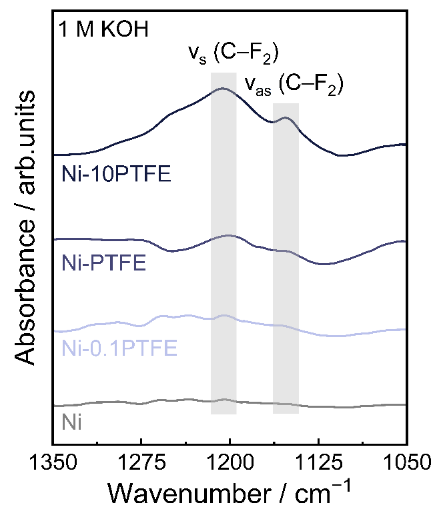


**Figure S11.** ATR-SEIRAS spectra of Ni-PTFE, Ni, Ni-0.1PTFE, and Ni-10PTFE. The peaks at 1200 cm^−1^ and 1150 cm^−1^ correspond to the symmetric and asymmetric stretching vibrations of C–F_2_, respectively^[14-16]^.


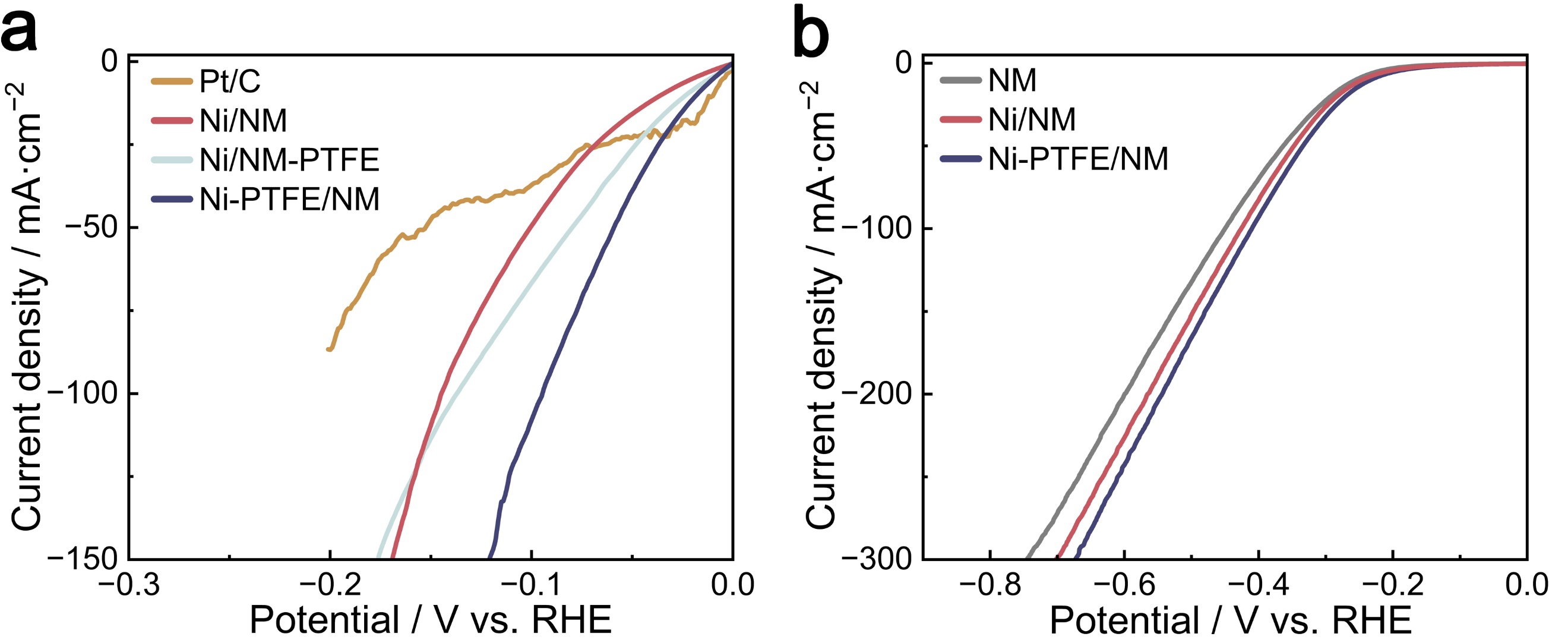


**Figure S12.** (a) HER polarization curves of Pt/C, Ni/NM-PTFE, Ni/NM, and Ni-PTFE/NM electrodes. (b) HER polarization curves of NM, Ni/NM, and Ni-PTFE/NM electrodes without IR compensation.


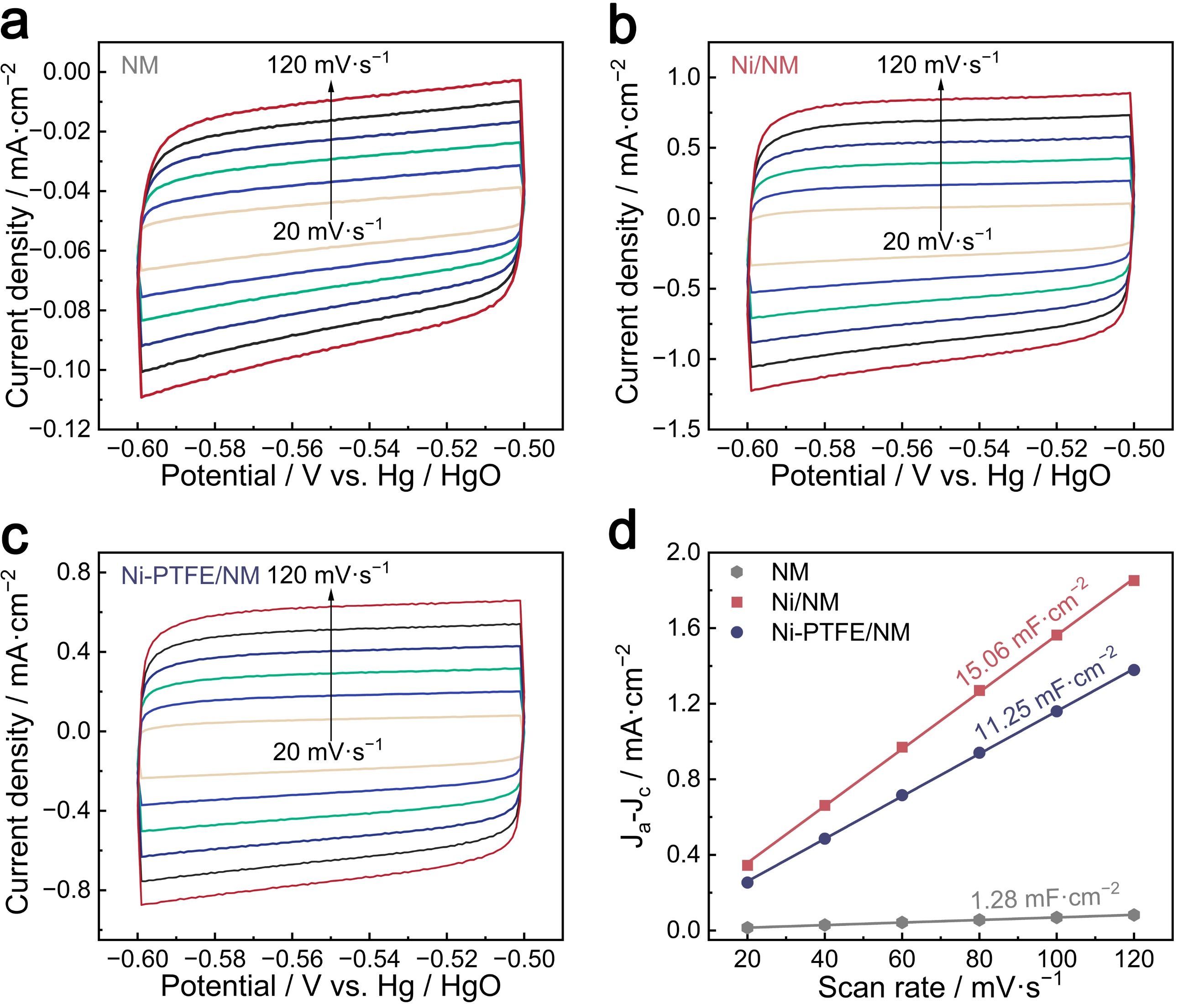


**Figure S13.** The CV curves of (a) NM, (b) Ni/NM, and (c) Ni-PTFE/NM electrodes. (d) Linear fitting of HER Δj vs. scan rates at −0.55 V vs. Hg/HgO.


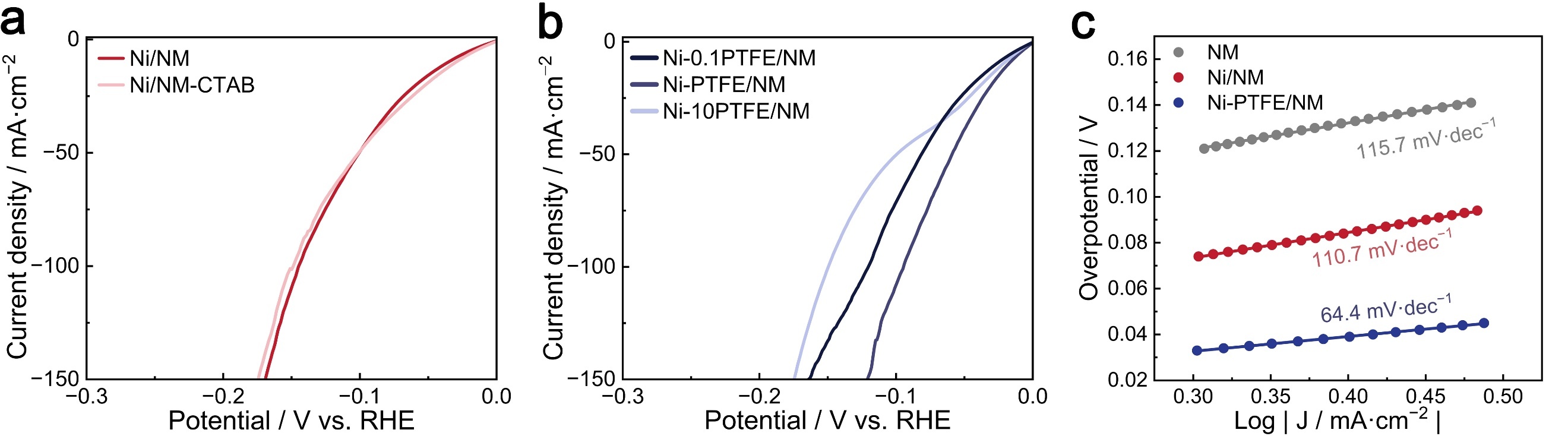


**Figure S14.** (a-b) HER polarization curves on the Ni/NM, Ni/NM-CTAB, Ni-PTFE/NM, Ni-0.1PTFE/NM and Ni-10PTFE/NM electrodes. (c) Slopes of the ECSA-normalized LSV curves of NM, Ni/NM, and Ni-PTFE/NM electrodes.


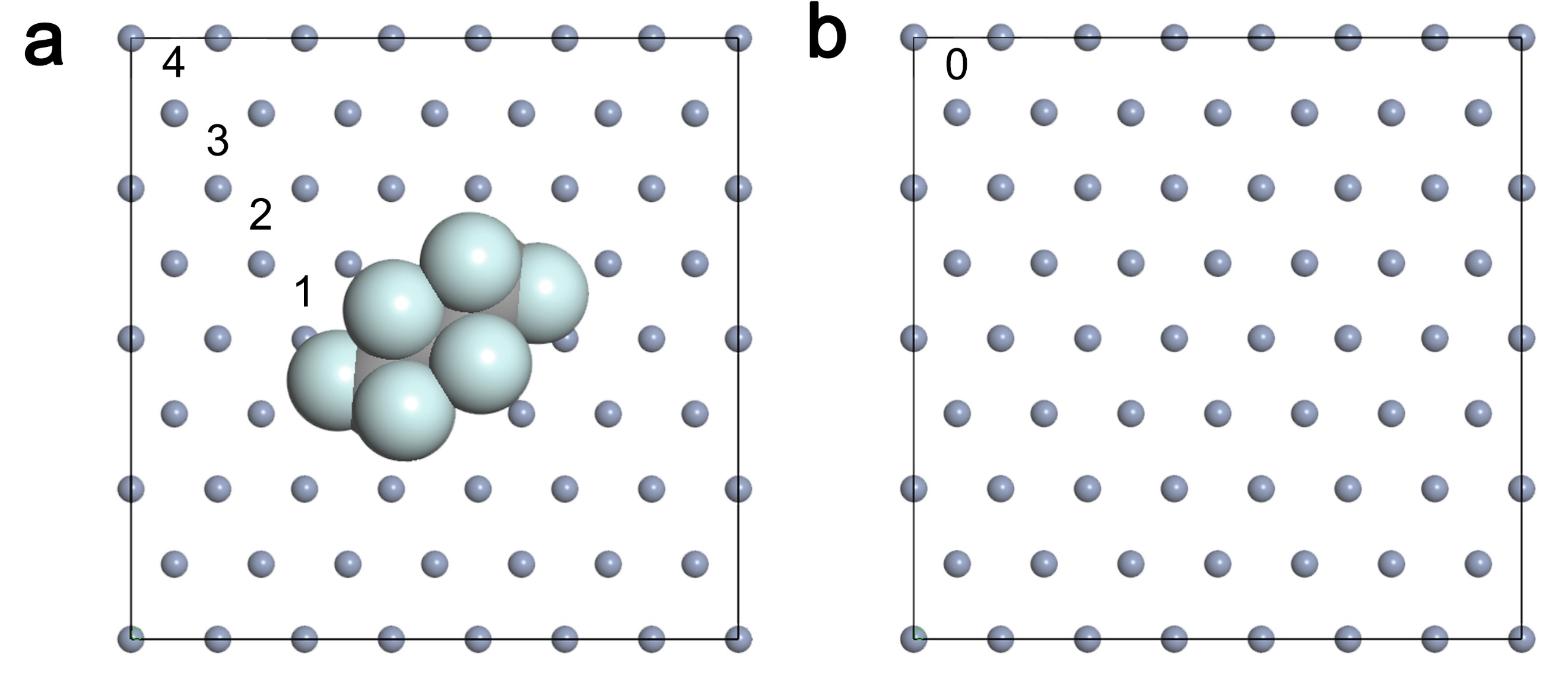


**Figure S15.** Top view of the atomic configuration of (a) Ni-PTFE and (b) Ni.


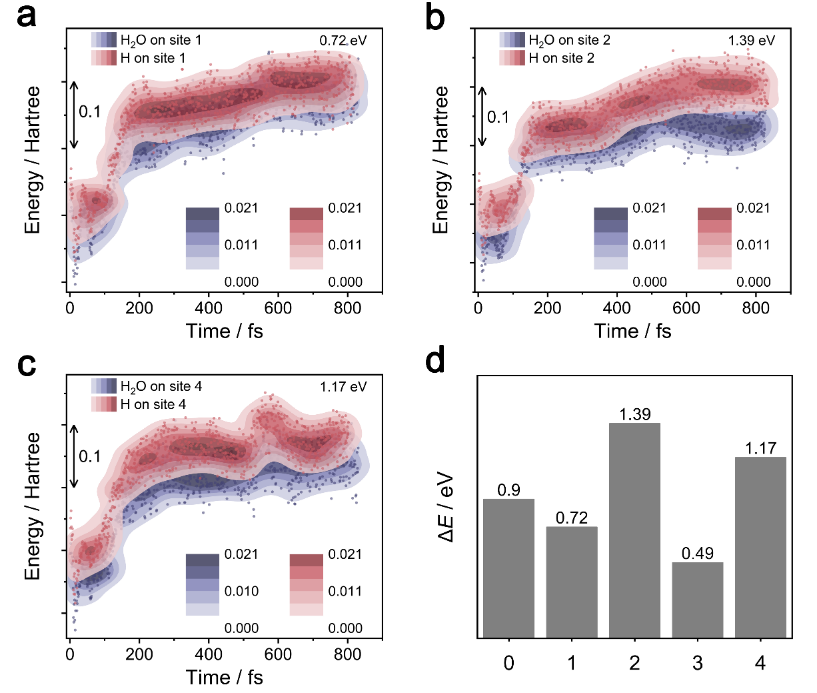


**Figure S16.** The adsorption energy distribution of H and H_2_O at (a) site 1, (b) site 2 and (c) site 4 of Ni-PTFE. (d) Bar chart of Δ*E*.


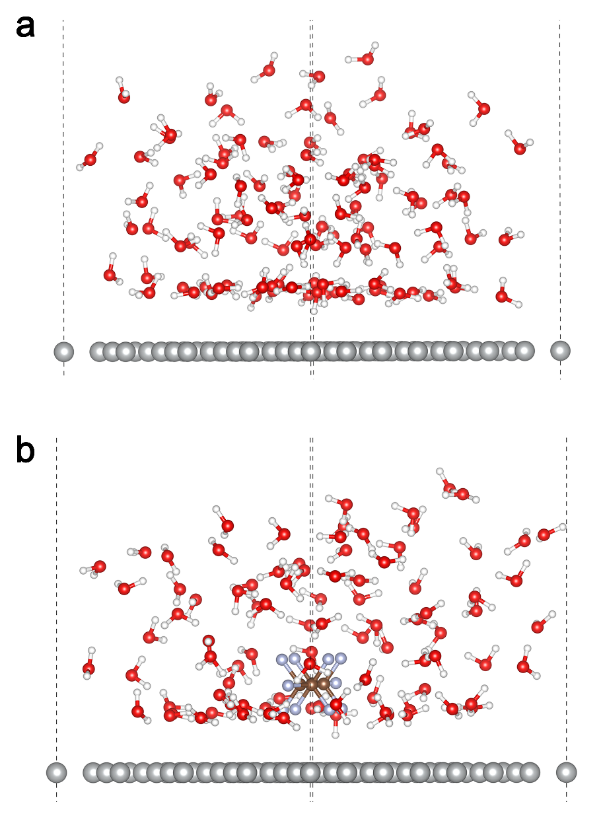


**Figure S17.** Representative front-view snapshots of the simulated interfacial structures on (a) Ni and (b) Ni-PTFE.


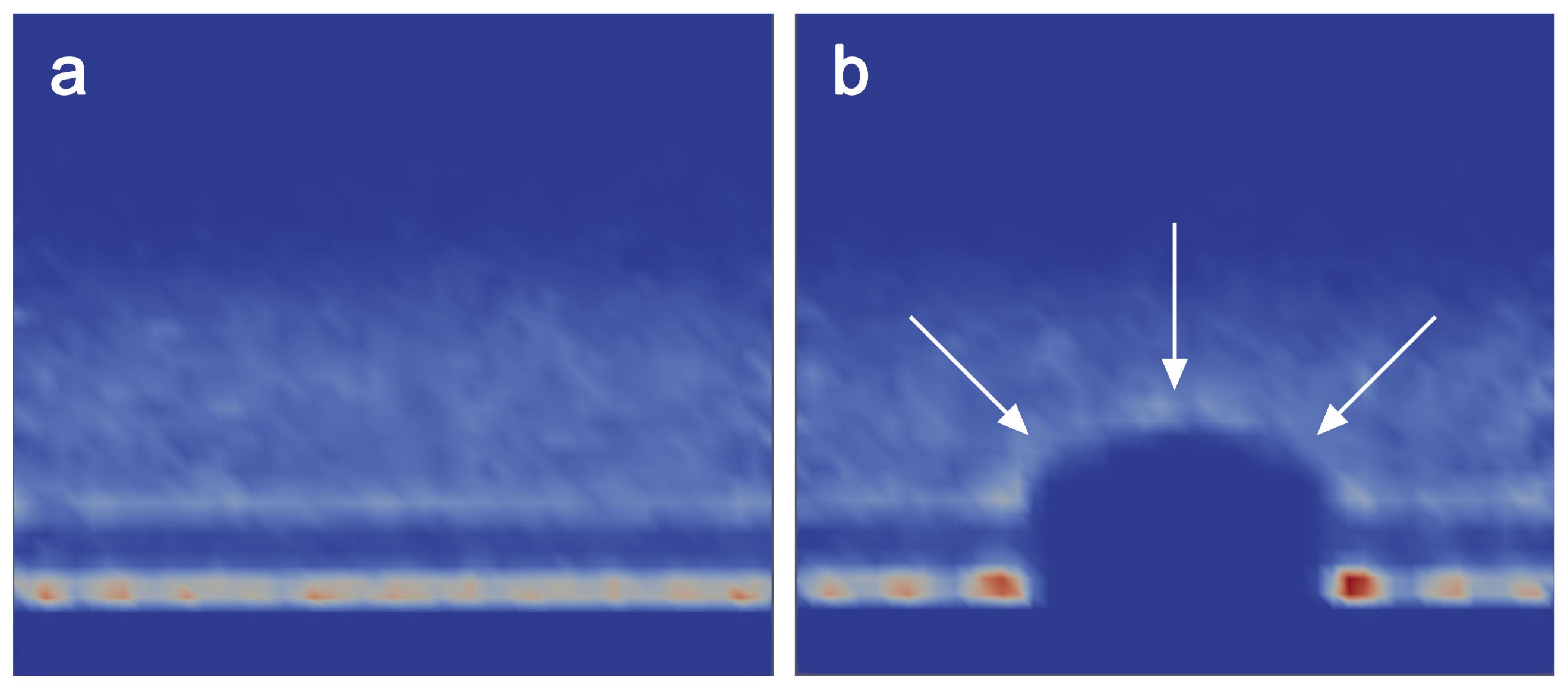


**Figure S18.** Water molecular number density distribution in the cross-sectional side view of (a) Ni and (b) Ni-PTFE.


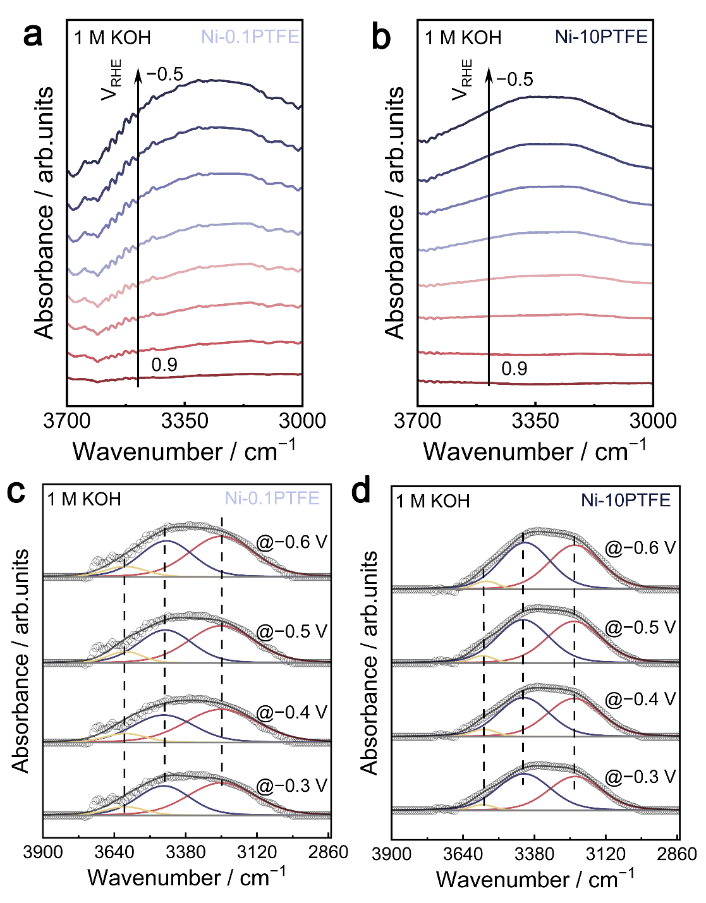


**Figure S19.** In situ ATR-SEIRAS spectra recorded during I-t curve the (a) Ni-0.1PTFE and (b) Ni-10PTFE potential from 0.9 to −0.5 V vs. RHE in 1 M KOH. Deconvoluted O–H stretching bands from in situ ATR-SEIRAS spectra of (c) Ni-0.1PTFE and (d) Ni-10PTFE electrodes at potentials from −0.3 to −0.6 V vs. RHE in 1 M KOH.


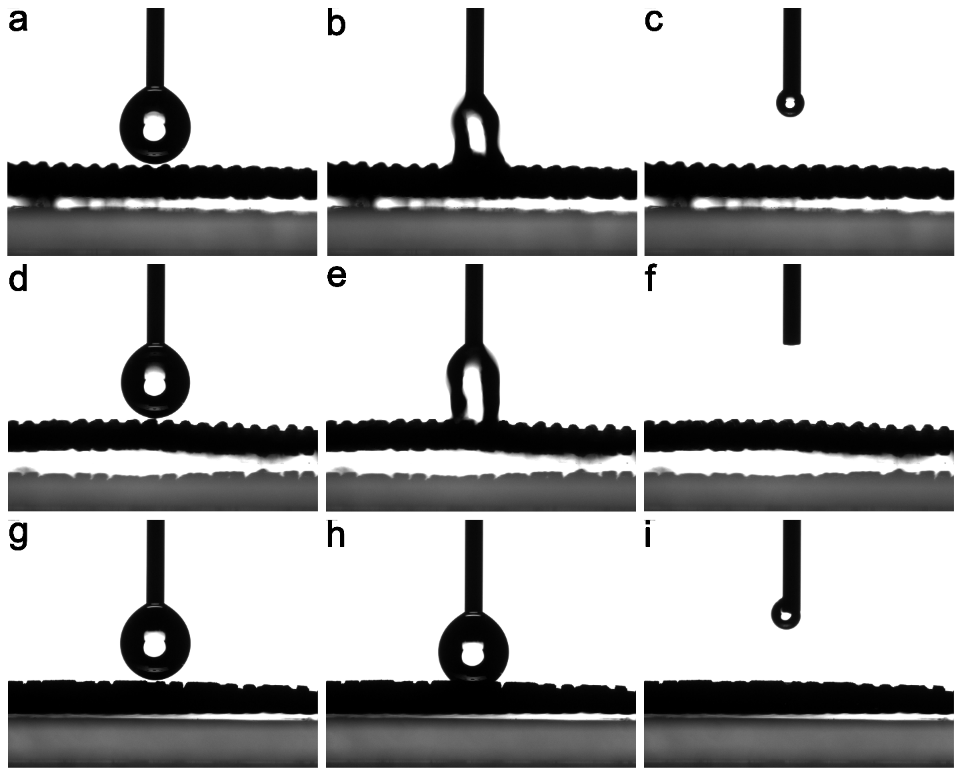


**Figure S20.** Wetting tests on (a-c) Ni/NM, (d-f) Ni-0.1PTFE/NM and (g-i) Ni-10PTFE/NM electrodes.


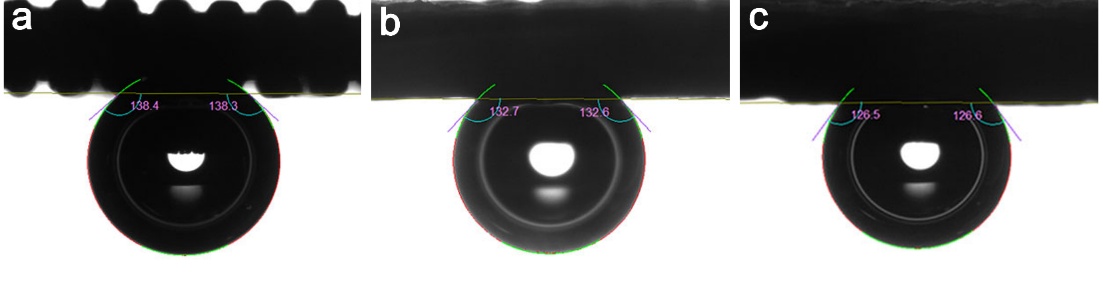


**Figure S21.** Underwater bubble CA tests on (a) Ni/NM, (b) Ni-0.1PTFE/NM and (c) Ni-10PTFE/NM electrodes.


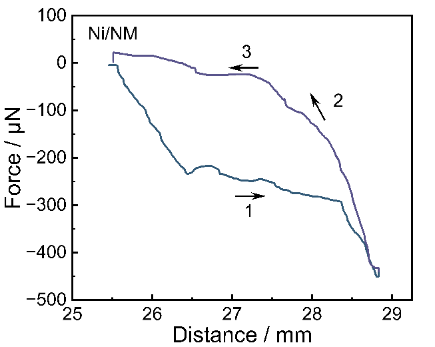


**Figure S22.** Adhesive forces measurements of the gas bubble on Ni/NM electrode.


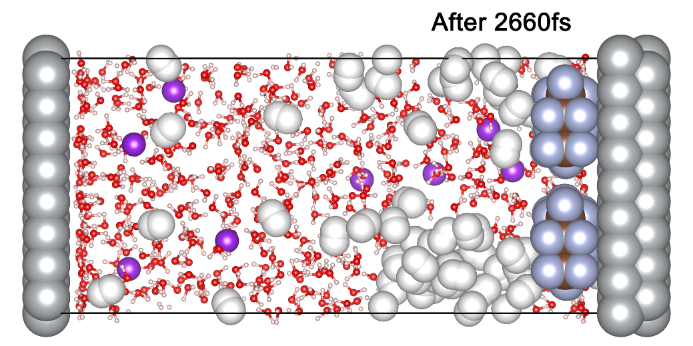


**Figure S23.** Snapshots of the *ab initio* MD simulated interfacial structure on the Ni-PTFE at the 2660 fs state.


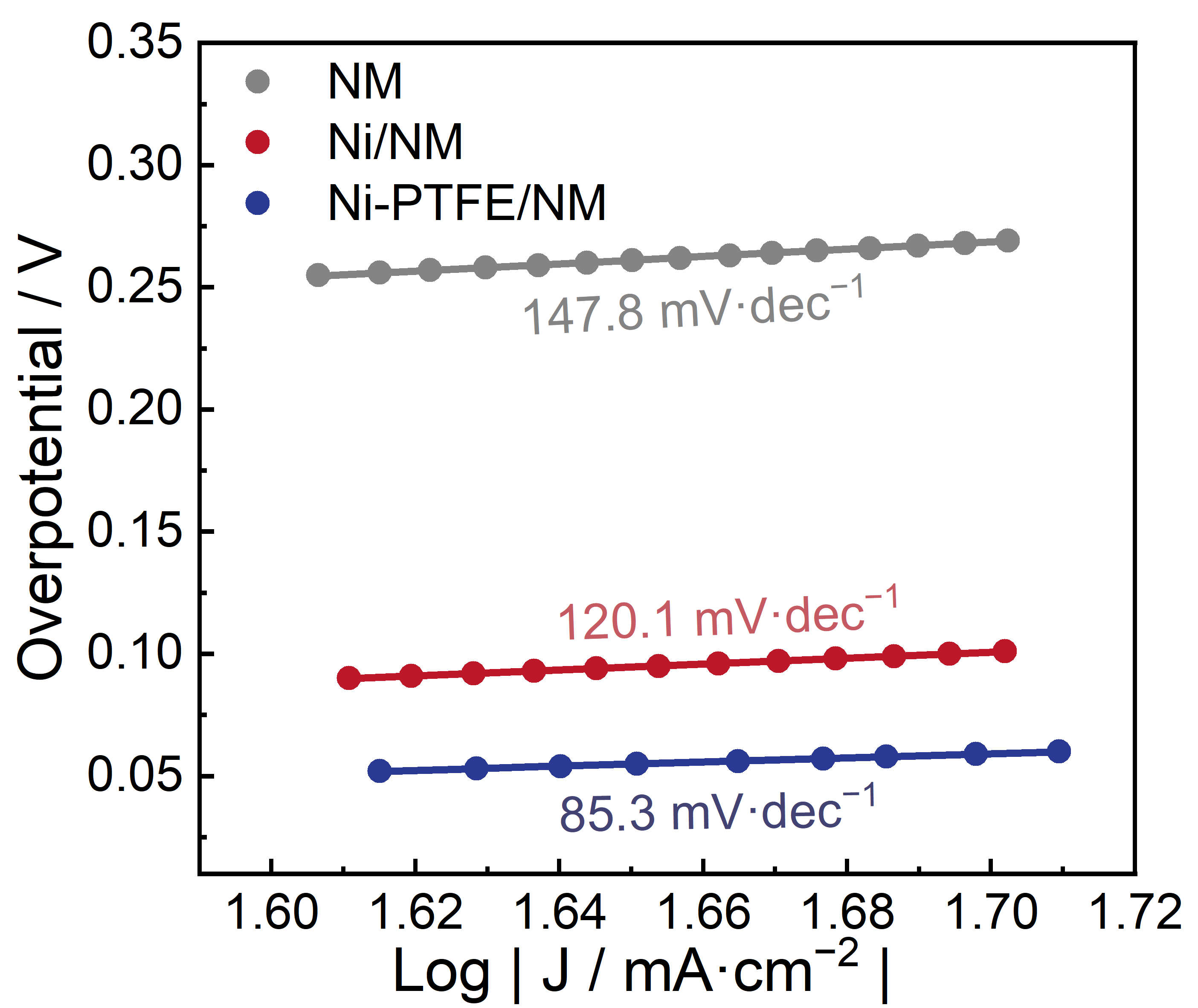


**Figure S24.** Slopes of LSV curves in the mass transport-limited region.


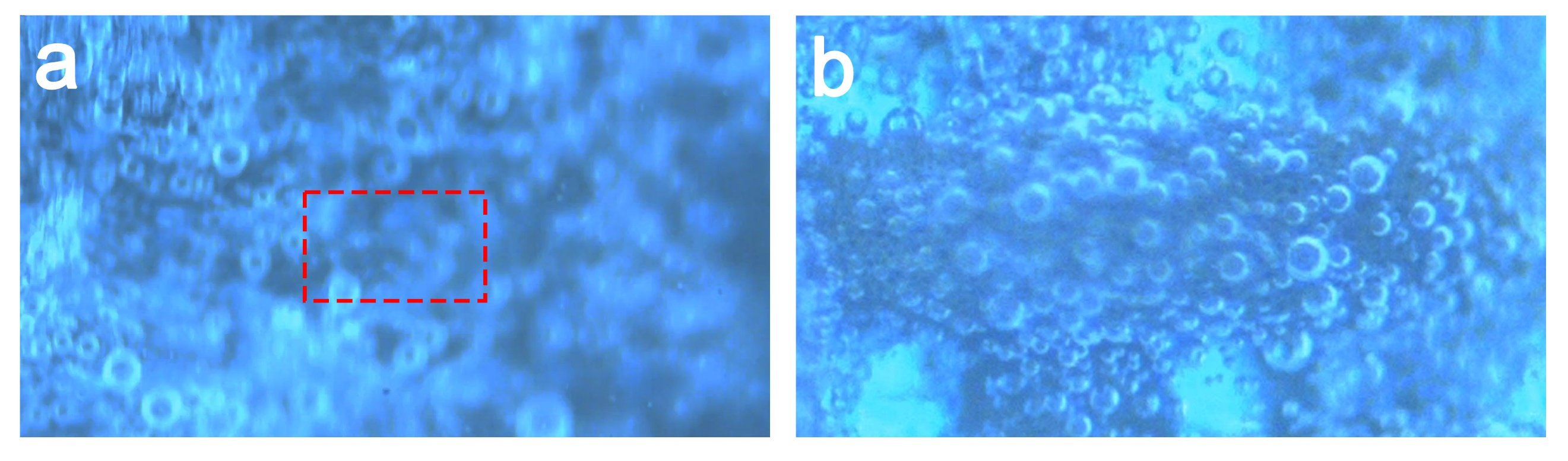


**Figure S25.** (a) Optical image of the Ni-PTFE/NM electrode surface under a current density of 500 mA·cm^−2^. (b) Optical image of the Ni-PTFE/NM electrode surface within a few seconds after the cessation of the applied potential.


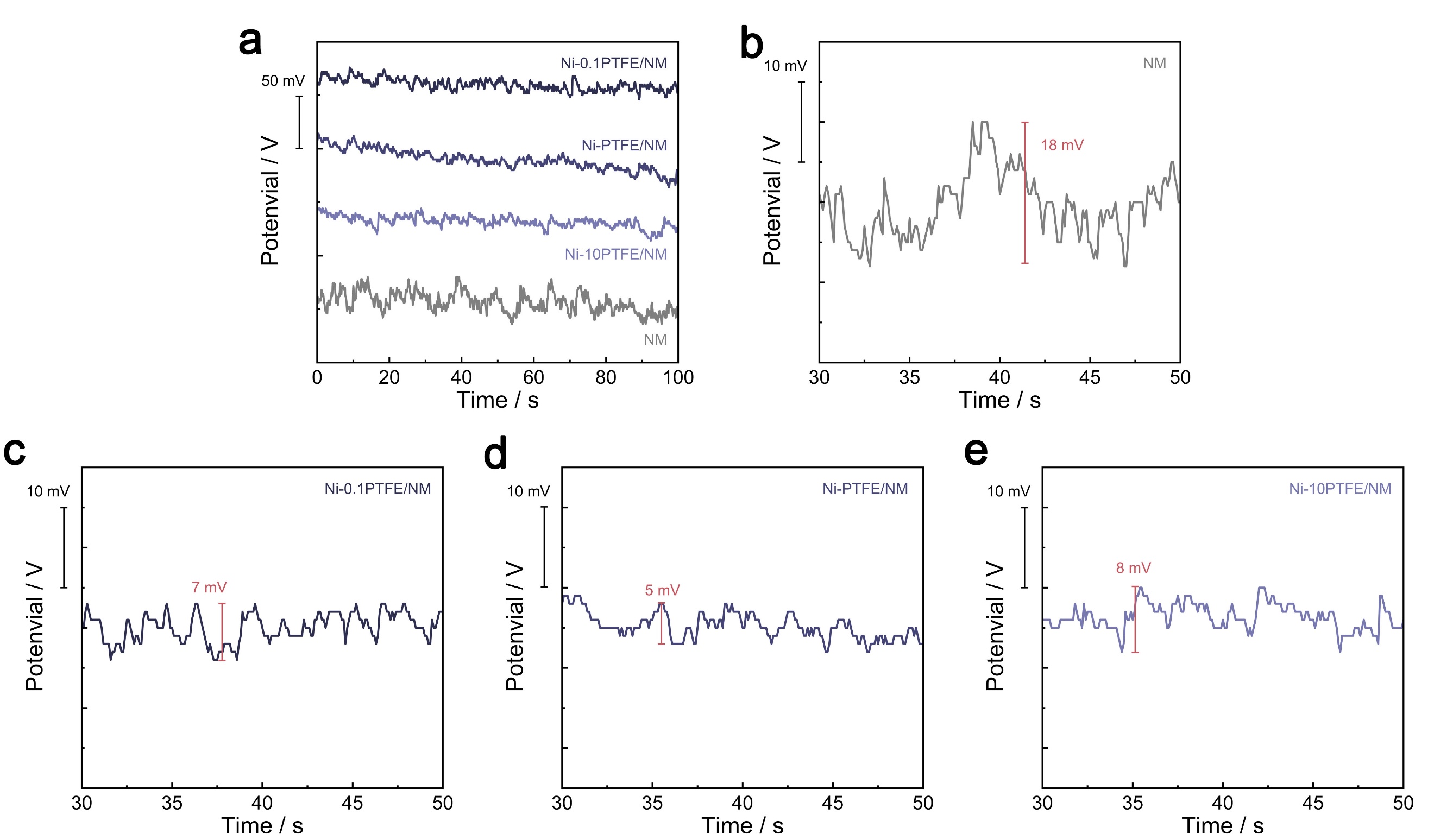


**Figure S26.** (a) Cp tests for HER of NM, Ni-0.1PTFE/NM, Ni-PTFE/NM and Ni-10PTFE/NM electrodes at 500 mA·cm^−2^. Enlarged view of selected CP curves for (b) NM, (c) Ni-0.1PTFE/NM, (d) Ni-PTFE/NM and (e) Ni-10PTFE/NM electrodes.


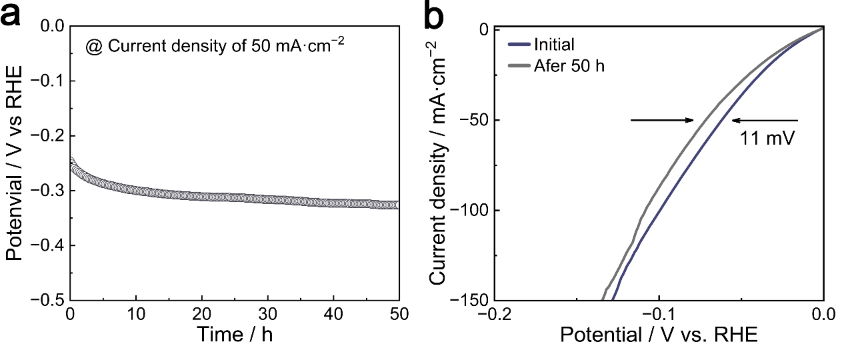


**Figure S27.** (a) CP test of Ni-PTFE/NM electrode for HER at 50 mA·cm^−2^ for 50 h. (b) HER polarization curves of Ni-PTFE/NM electrode before and after the 50 h CP test.


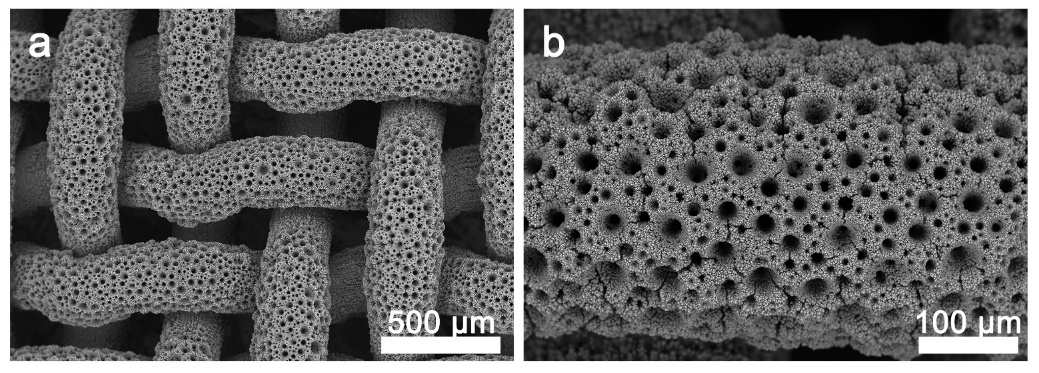


**Figure S28.** SEM images of the Ni-PTFE/NM electrode after HER stability test.


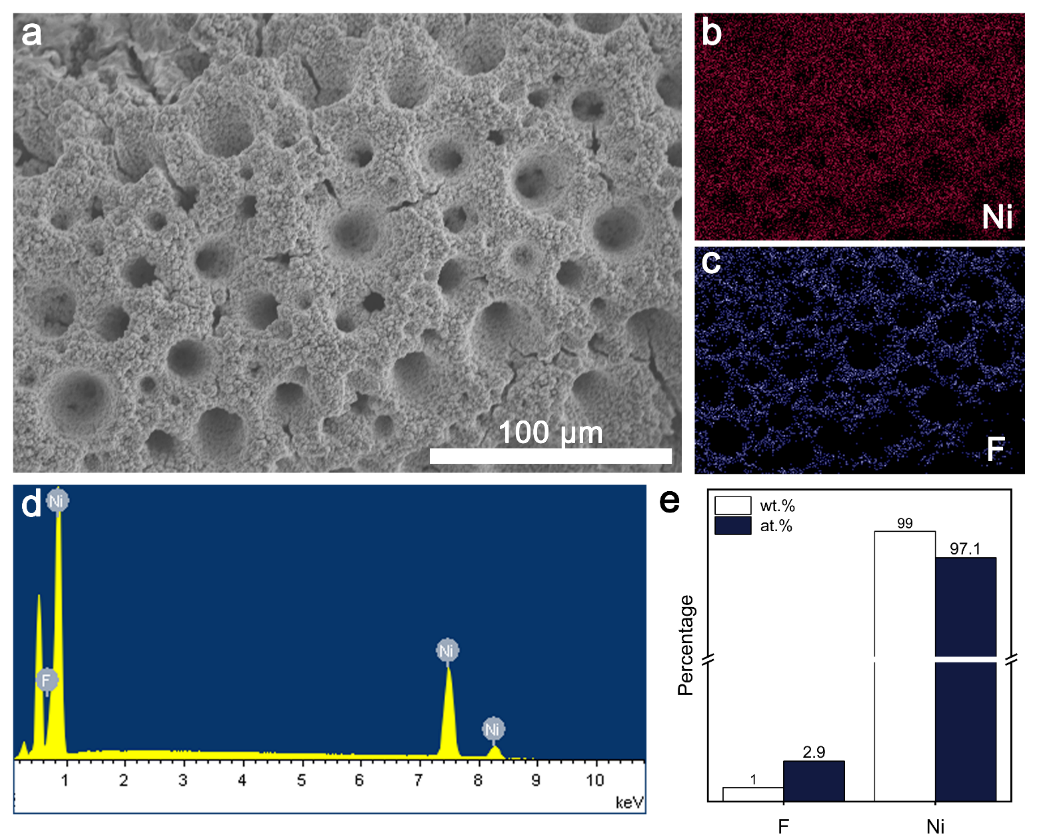


**Figure S29.** SEM of Ni-PTFE/NM electrode after HER stability test. (a) SEM image. EDS mapping of (b) Ni and (c) F elements. (d) EDS spectrum and (e) elemental composition bar chart.


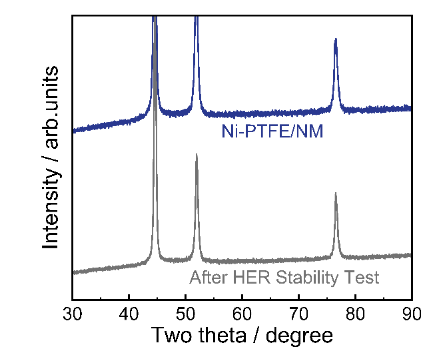


**Figure S30.** The XRD patterns of the Ni-PTFE/NM electrode after HER stability test.


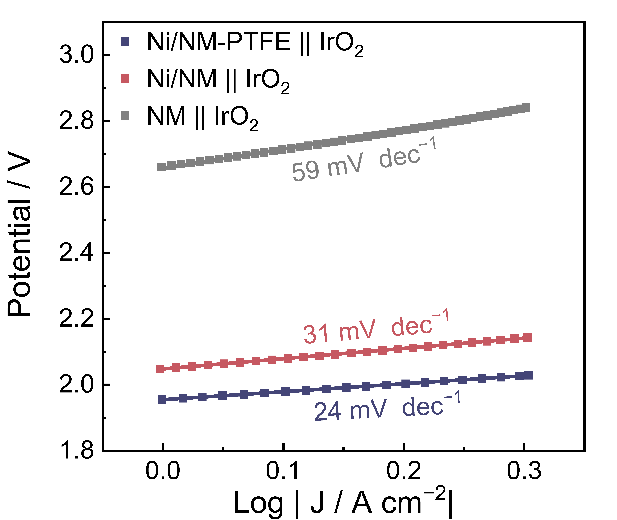


**Figure S31.** Slope of LSV curves in the current density range of 1-2 A·cm^−2^.

**Table S1.** The gas channel diameter of the electrodes.

| Electrode | Ni/NM | Ni-0.1PTFE/NM | Ni-PTFE/NM | Ni-10PTFE/NM |
| --- | --- | --- | --- | --- |
| Size | 12.7 μm | 12.9 μm | 16.9 μm | 33.7 μm |

**Table S2.** Impedance fitting via equivalent circuit modeling^[17, 18]^.

| Electrode | R_s_ | R_ct_ | CPE-T | CPE-P |
| --- | --- | --- | --- | --- |
| NM | 0.93 | 44.7 | 0.0006 | 0.83 |
| Ni/NM | 0.96 | 4.1 | 0.037 | 0.87 |
| Ni-PTFE/NM | 0.94 | 3.1 | 0.039 | 0.90 |

**Table S3.** Comparison of overpotential (at 10 mA·cm^−2^) and Tafel slope for different electrodes.

| Electrode | 10 mA·cm^−2^ | mV·dec^−1^ | Ref. |
| --- | --- | --- | --- |
| Ni-PTFE/NM | 19 | 30 | This work |
| Ni_3_Se_4_–Ni_3_N | 60 | 51 | ^[19]^ |
| W_1_O/NiS | 76 | 42 | ^[20]^ |
| Fe-CPNCF | 19 | 57 | ^[21]^ |
| IrNi/MWCNTs | 63 | 49 | ^[22]^ |
| Ni/MoBT_x_ | 32 | 27 | ^[23]^ |
| NiSe-90 | 205 | 82 | ^[24]^ |
| MoN@NiO-300 | 55 | 65 | ^[25]^ |
| Ru_1_–1T-MoS_2_/CC | 48 | 44 | ^[26]^ |
| Fe_d_-NiS_2_/MXene | 148 | 85 | ^[27]^ |
| MoC/Co/NCNT | 33 | 47 | ^[28]^ |
| Ni_3_N/NF | 189 | 92 | ^[29]^ |
| R-NiMoO_4_@NiS_2_/NF | 44 | 63 | ^[30]^ |
| CoNi/CoNiMo_3_O_8_ | 22 | 60 | ^[31]^ |
| Co/Co_2_VO_4_@MoO_2_ | 52 | 80 | ^[32]^ |
| WB_2_@WS_2_ | 170 | 56 | ^[33]^ |
| Ni-WNO | 35 | 32 | ^[34]^ |
| Al_P_-MoO_2_@Mo_2_C | 98 | 45 | ^[35]^ |

**Table S4.** SEIRAS peak area ratio.

| Electrode | Adlayer water | H_2_O_(above-gap)_ | H_2_O_(gap)_ |
| --- | --- | --- | --- |
| Ni | 55.0% | 36.6% | 8.4% |
| Ni-0.1PTFE | 52.8% | 38.6% | 8.6% |
| Ni-PTFE | 40.1% | 50.5% | 9.4% |
| Ni-10PTFE | 47.6% | 48.4% | 4.0% |

**References**

1. A. K. Rappe, C. J. Casewit, K. S. Colwell, W. A. Goddard, III, W. M. Skiff, *J. Am. Chem. Soc.* **1992**, *114*, 10024-10035.

2. A. K. Rappe, W. A. Goddard, III, *The Journal of Physical Chemistry* **1991**, *95*, 3358-3363.

3. A. A. Samoletov, C. P. Dettmann, M. A. J. Chaplain, *Journal of Statistical Physics* **2007**, *128*, 1321-1336.

4. N. Metropolis, A. W. Rosenbluth, M. N. Rosenbluth, A. H. Teller, E. Teller, *The Journal of Chemical Physics* **1953**, *21*, 1087-1092.

5. J. Hutter, M. Iannuzzi, F. Schiffmann, J. VandeVondele, **2014**, *4*, 15-25.

6. J. P. Perdew, K. Burke, M. Ernzerhof, *Physical Review Letters* **1996**, *77*, 3865-3868.

7. S. Grimme, *Journal of Computational Chemistry* **2006**, *27*, 1787-1799.

8. S. Goedecker, M. Teter, J. Hutter, *Physical Review B* **1996**, *54*, 1703-1710.

9. C. Hartwigsen, S. Goedecker, J. Hutter, *Physical Review B* **1998**, *58*, 3641-3662.

10. J. VandeVondele, J. Hutter, *The Journal of Chemical Physics* **2007**, *127*.

11. G. Bussi, D. Donadio, M. Parrinello, *The Journal of Chemical Physics* **2007**, *126*.

12. T. Lu, F. Chen, *Journal of Computational Chemistry* **2012**, *33*, 580-592.

13. T. Lu, *The Journal of Chemical Physics* **2024**, *161*.

14. J.-L. Fu, Y. Liu, Y.-M. Chen, H. Zhang, J.-P. Qu, Y.-B. Kang, *Angew. Chem. Int. Ed.* **2025**, *64*, e202422043.

15. J. Piwowarczyk, R. Jędrzejewski, D. Moszyński, K. Kwiatkowski, A. Niemczyk, J. Baranowska, in *Polymers, Vol. 11*, 2019, p. 1629.

16. B. Deng, X. He, P. Du, W. Zhao, Y. Long, Z. Zhang, H. Liu, K. Huang, H. Wu, *Adv. Sci.* **2024**, *11*, 2408544.

17. B. Wang, M. Lu, D. Chen, Q. Zhang, W. Wang, Y. Kang, Z. Fang, G. Pang, S. Feng, *J. Mater. Chem. A* **2021**, *9*, 13562–13569.

18. G. Li, L. Anderson, Y. Chen, M. Pan, P.-Y. Abel Chuang, *Sustainable Energy Fuels* **2018**, *2*, 237–251.

19. D. D. Alemayehu, M.-C. Tsai, M.-H. Tsai, C.-C. Yang, C.-C. Chang, C.-Y. Chang, E. A. Moges, K. Lakshmanan, Y. Nikodimos, W.-N. Su, C.-H. Wang, B. J. Hwang, *J. Am. Chem. Soc.* **2025**, *147*, 16047–16059.

20. W.-G. Cui, X. Ren, S. Wang, Y. Zhang, Z. Li, K. Wang, F. Gao, Z. Shen, Y. Liu, X. Wang, Z. Wu, Y. Yang, D. Wang, H. Pan, *Adv. Energy Mater.* **2025**, *15*, e03257.

21. L. wang, X.-W. Lv, H.-Y. Wang, J.-T. Ren, Y. Feng, M. Sun, Z.-Y. Yuan, *Adv. Energy Mater.* **2025**, *15*, e04036.

22. G. Zhang, S. Qi, H. Wang, X. Lei, Z. Zhang, W. Liu, X. Liu, Y. Zhao, L. Yang, W. Ma, W. Fan, X. Zong, *Adv. Funct. Mater.* **2025**, *n/a*, e17113.

23. H.-L. Su, W. Liao, S.-X. Mo, H. Wang, Y. Cao, H. Yu, H.-F. Wang, *Adv. Funct. Mater.* **2025**, *n/a*, e18373.

24. X. Ding, D. Liu, A. wang, P. Zhang, F. E. Oropeza, P. Zhao, G. Gorni, M. Barawi, V. de la Peña O'Shea, R. Wu, K. H. L. Zhang, *Adv. Funct. Mater. n/a*, e21566.

25. D. Feng, P. Wang, B. Ma, X. Zhao, Y. Chen, *Appl. Catal., B* **2025**, *374*, 125373.

26. Z. Wang, X. Zhao, D. Liu, X. Zhang, Y. Gao, H. Wang, *Appl. Catal., B* **2026**, *382*, 126029.

27. Y. Zhao, Y. Zhu, C. Xi, K. Hu, S. Han, J. Jiang, *Appl. Catal., B* **2025**, *378*, 125611.

28. C. Liu, X. Cao, L. Chen, Q. Wang, B. Zhang, C. Liang, Y. M. Lam, L. Wang, *ACS Catal.* **2025**, *15*, 14983–14995.

29. C. Tyagi, P. Basera, C. Lagrost, V. Bouquet, F. Tessier, M.-C. Jullien, B. Fabre, *ACS Catal.* **2025**, *15*, 3823–3835.

30. X. Luo, H. Zhao, L. Wang, X. Tan, L. Guo, L. Jiang, M. Wang, Z. Tao, S. Mu, *Nano Energy* **2026**, *147*, 111601.

31. H. Hao, J. Wang, Z. Wang, J. Li, Z. Jian, B. Liu, S. Shen, L. Xu, Z. Lv, B. Wei, *Nano Energy* **2025**, *141*, 111125.

32. H. Li, Y. Xiao, Z. Wang, B. Yao, S. Yuan, Q. Feng, M. Cao, Y. Wang, *Nano Energy* **2025**, *141*, 111087.

33. M. Wang, C. Yan, T. Liu, S. Wang, Z. Liao, F. Guo, Q. Wang, Z. Li, G. Wang, *Angew. Chem. Int. Ed.* **2025**, *64*, e202425657.

34. H. Fu, Y. Wu, L. Wang, Z. Qin, L. Chen, Y. Li, K. Shen, *Angew. Chem. Int. Ed.* **2025**, *64*, e18043.

35. Z. Chen, M. Yang, Y. Li, W. Gong, J. Wang, T. Liu, C. Zhang, S. Hou, G. Yang, H. Li, Y. Jin, C. Zhang, Z. Tian, F. Meng, Y. Cui, *Nat. Commun.* **2025**, *16*, 418.
